# A Description of Personal Health Information Management Work With a Spotlight on the Practices of Older Adults: Qualitative e-Delphi Study With Professional Organizers

**DOI:** 10.2196/42330

**Published:** 2023-03-31

**Authors:** Deborah E Seale, Cynthia M LeRouge, Malgorzata Kolotylo-Kulkarni

**Affiliations:** 1 Department of Public Health College of Health Sciences Des Moines University Des Moines, IA United States; 2 Department of Information Systems & Business Analytics College of Business Florida International University Miami, FL United States; 3 Department of Information Management & Business Analytics Zimpleman College of Business Drake University Des Moines, IA United States

**Keywords:** patient work system, consumer health informatics, personal health information management, PHIM, patient participation, medical informatics, information management

## Abstract

**Background:**

Personal health information (PHI) is created on behalf of and by health care consumers to support their care and wellness. Available tools designed to support PHI management (PHIM) remain insufficient. A comprehensive understanding of PHIM work is required, particularly for older adults, to offer more effective PHIM tools and support.

**Objective:**

The primary objective of this study was to use the Patient Work System model to provide a holistic description of PHIM work from the perspective of professional organizers with experience assisting health care consumers, including older adults, in managing their PHI. A secondary objective was to examine how factors associated with 4 Patient Work System components (person, tasks, tools and technologies, and context) interact to support or compromise PHIM work performance.

**Methods:**

A modified e‐Delphi methodology was used to complete 3 web-based rounds of open-ended questions and obtain consensus among a panel of 16 experts in professional organizing. Data were collected between April and December 2017. The Patient Work System model was used as a coding schema and guided the interpretation of findings during the analysis.

**Results:**

The PHIM work of adults who sought assistance focused on the tasks of acquiring, organizing, and storing 3 classifications of PHI (medical, financial, and reference) and then processing, reconciling, and storing the medical and financial classifications to tend to their health, health care, and health finances. We also found that the complexities of PHI and PHIM-related work often exceeded the abilities and willingness of those who sought assistance. A total of 6 factors contributed to the complexity of PHIM work. The misalignment of these factors was found to increase the PHIM workload, particularly for older adults. The life changes that often accompanied aging, coupled with obscure and fragmented health care provider- and insurer-generated PHI, created the need for much PHIM work. Acquiring and integrating obscure and fragmented PHI, detecting and reconciling PHI discrepancies, and protecting PHI held by health care consumers were among the most burdensome tasks, especially for older adults. Consequently, personal stakeholders (paid and unpaid) were called upon or voluntarily stepped in to assist with PHIM work.

**Conclusions:**

Streamlining and automating 2 of the most common and burdensome PHIM undertakings could drastically reduce health care consumers’ PHIM workload: developing and maintaining accurate current and past health summaries and tracking medical bills and insurance claims to reconcile discrepancies. Other improvements that hold promise are the simplification and standardization of commonly used financial and medical PHI; standardization and automation of commonly used PHI acquisition interfaces; and provision of secure, Health Insurance Portability and Accountability Act (HIPAA)–certified PHI tools and technologies that control multiperson access for PHI stored by health care consumers in electronic and paper formats.

## Introduction

### Background

Health care consumers regularly encounter and track personal health information (PHI) in various forms. For example, health care consumers may track their activity levels using a wellness tool, view their laboratory results, or receive medical bills. Health care consumers of all ages may be involved in or exhibit needs for PHI management (PHIM). However, PHIM is particularly important and prevalent among older adults. Older adults are likely to be undergoing, or anticipating, life-stage transitions, such as care giving (for parents, spouses, children, and grandchildren), retirement, starting Medicare, and encountering health issues associated with aging. Consequently, these individuals often find themselves in situations requiring a substantial need for PHIM for themselves and their families.

Effective PHIM can be beneficial as it facilitates patients’ self-care [1], encourages them to contribute to their health care [2], and improves patient-provider communication [2]. Unfortunately, PHIM is multifaceted and complex. The vast amount of health information generated and the growing need to manage it can be overwhelming, particularly for older adults and those with chronic health issues. Furthermore, the tasks associated with handling PHI often involve multiple stakeholders, including close relatives assisting or caring for the person, such as a spouse or an adult child [3]. PHI stakeholders also include the person’s health care providers [3] defined herein as “persons who are trained and licensed to give health care” or “places that are licensed to give health care” [4].

PHI is also frequently scattered among health care providers. With little support for an efficient and effortless transfer of information, health care consumers struggle with the burden of PHI coordination [5]. Tools that support PHIM, such as patient portals, have been shown to be insufficient with respect to PHIM challenges, such as the fragmentation of PHI [6,7], and recommendations for tools to reduce fragmentation have recently been raised [8]. The physical environment also plays a vital role. For instance, individuals need to choose the most optimal storage location for PHI so that it is easily accessible in an emergency [9]. In addition, research has shown that older adults are less likely to use consumer health informatics technology to manage their health and health care [10-13]. Hence, we continue to face numerous unanswered questions regarding PHIM.

**Figure 1:**
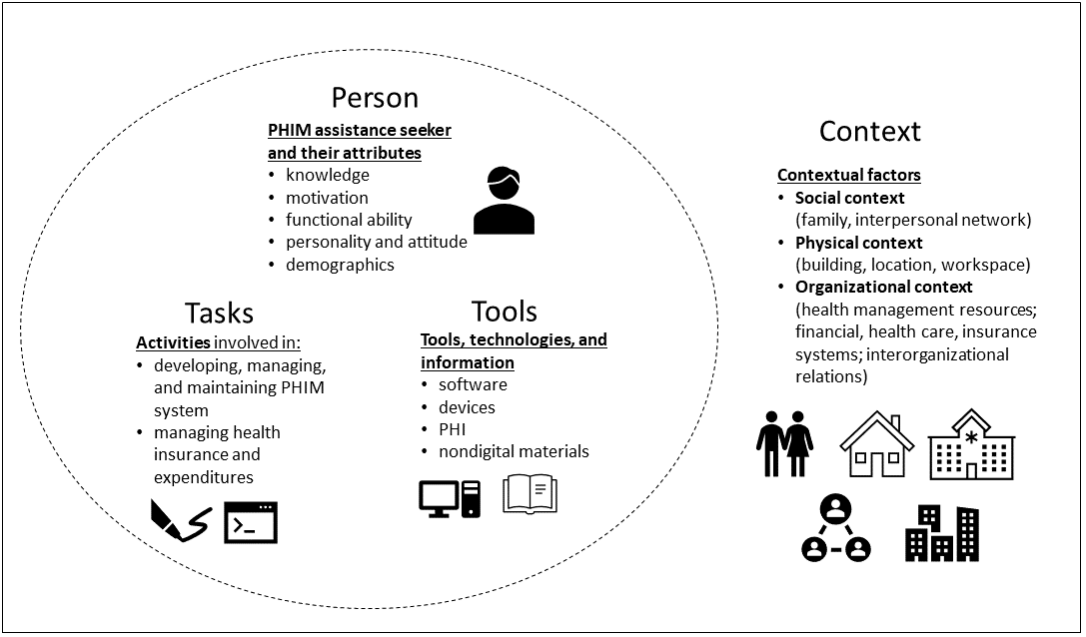
Patient Work System conceptual model adapted from prior framework components [16-19]. PHI: personal health information; PHIM: personal health information management.

At its core, PHI is an element of a work system that aims to sustain and promote health. A system is a set of diverse interacting elements within an environment; the relationships or interactions between elements are often as important as the elements themselves in determining the behavior of the system [14,15]. For this study, we used a conceptual framework known as the Patient Work System to holistically describe PHIM as a work system. Figure 1 delineates the Patient Work System model as defined in this paper, consisting of 4 components. These components include the person engaged in PHIM, tasks carried out, tools used, and the context surrounding PHIM. The model was adapted based on prior frameworks [16-18] in a deductive-inductive fashion to incorporate both existing research [16-18] and the findings of this study.

The first component, person, refers to the characteristics of the person receiving PHIM assistance and how these characteristics influence PHIM work [19]. Person characteristics may include “individual knowledge, motivation, functional ability, personality and attitude, and demographic factors” [19].

The second patient work component, tasks, refers to activities involved in developing, managing, and maintaining a PHIM system, including managing health insurances and financial expenditures. Tasks include properties, such as difficulty, timing, and complexity. Tools and technologies, the third component of the Patient Work System, refers to artifacts, including software, devices, PHI, and nondigital materials used to support PHIM tasks carried out by health care consumers [17,20]. Relevant properties include accessibility, availability, usability, and effectiveness of PHIM tools. The fourth component, context, focuses on 3 distinct contextual dimensions [21] in the PHIM assistance seekers’ environment:

- Social environment, such as family and other interpersonal networks, at the household and community levels
- Physical or built environment, such as weather and indoor or outdoor climate; physical distance, layout, and surface; workspace; location; lighting; organization; and access to utilities
- Organizational environments, such as resources for health management (including health information systems [HISs]), financial, health care, and insurance systems (including HIS), interorganizational communication, and information sharing (including through HIS)

The Patient Work System has been a widely adopted framework and has been applied in studies to understand patient self-care [19,21,22]. It has also been adopted in PHIM research, including in a literature review [3] and empirical studies that focused on PHIM as a work system [8,23,24].

Research to date adopting the Patient Work System as a lens to examine PHIM [5,8,23-26] has consistently considered 3 of the patient work model components (person or persons, tasks, and tools and technologies), describing the person or persons (the patient, their caregivers, and health care professionals) carrying out the tasks and using the tools to do the work. In contrast, the number, definitions, and specifications of the context elements describing PHIM work have varied across the studies.

There has been inconsistency in how the organizational element has been considered. For instance, some studies define it in terms of personal relationships [5] or the household [26]. The conceptualization and focus of the context elements also differ among studies. Variations include examining PHIM within “organizational, social, and physical contexts” [24], within the “sociotechnical” system [23], or within the “social organization and physical environment” of the patient’s home [8]. Thus, there is a need for research to provide a sound investigation describing the context of PHIM. Considering the differentiation in how these elements have been perceived in the literature and their systems-based nature, it may be argued that contextual elements should be examined individually as well as holistically. This also underscores the need to further develop and specify the nature and definition of the contextual elements (similar calls were made by Holden et al [21] for patient self-care).

In addition, by considering individual work system components, researchers [17-19,23,27] argue for understanding how the components of the model interact (or fit) to influence patient work. It is through the interactions of person, tasks, tools, and context that PHI is managed—that the PHI work is done. It has been argued that a good fit between the elements of the work system can drive positive outcomes of work activity, whereas a poor fit can lead to declines in safety, efficiency, and effectiveness [17]. Understanding work activity and the burden of work on health care consumers will inform our understanding of health IT (HIT) development, health care provider practices, and the policies needed to relieve consumer burden and encourage engagement.

Thus far, component interactions have been addressed in studies of health behaviors. For instance, an examination of the interaction among the factors of the patient’s care environment, including clinical and social elements, has revealed the extent and intensity of burdens that patients have to deal with—related to illness (such as pain), treatment, and workload (demands in their lives, such as daily obligations including job or family) [28]. In addition, considering the concept of patient burden in particular, research has encouraged further studies that would enhance our understanding of the complex array of factors that play a role in patient burden in their entirety [28] and have recognized it as an important research area [27]. Therefore, an analysis of the interaction among the elements of the work system could reveal the burden experienced by patients associated with PHIM.

Furthermore, a holistic consideration of the work system elements, with an acknowledgment of the interactions among them, is important for the design and development of PHIM technologies. An omission of any of the model components or their interactions inhibits advancement in model formulation and problem definition as well as actions for the improvement and innovation of technological solutions [29]. A recent popular series of publications of mini-cases, HIT or Miss [30], is replete with stories of technically functional technology misaligned with the targeted user, context, or intended purpose. Indeed, silos of perspective can often hinder innovation and the emergence of HIT innovation and holistic results that are needed to address key health issues with the assistance of technology. Therefore, for HIT system developers, health care providers, and policy makers to assist health care consumers in their journey to become engaged and informed, a thorough and holistic understanding of PHIM work is needed.

The Patient Work System model incorporates the work of patients, their caregivers, family members, nonprofessionals, and other health care professionals [27]. In addition, a growing number of experts on the periphery of health care assist patients in navigating the health care space. Examples include peer or health coaches [31-34], advisers (eg, as part of Patient and Family Advisory Clinics) [35], and navigators [36]. Professional organizers also help patients in managing their PHI. Professional organizers specialize in organizing personal spaces and objects, including home offices and personal information [37]. The inclusion of PHIM work in the organizing efforts of professional organizers often naturally evolves from their ongoing work with clients (personal communication). Therefore, in this study, organizers serve as key informants, that is, knowledgeable observers and assistance providers for PHIM work. To the best of our knowledge, this is the first study to describe PHIM work from the perspective of professionals with experience in assisting health care consumers with PHIM work.

### Objectives

In this study, we aimed to better understand the PHIM work system (person, task, tools, and context) from the perspective of experts that assist health care consumers with PHIM. Specifically, the first objective was to describe PHIM work and explore the attributes of each of the components of the PHIM work system in detail: the person who is the focus of PHIM, tasks carried out, tools used, and the context surrounding the work (including organizational, social, and physical dimensions). The secondary objective was to explicate the interaction among the components of the system—to gauge to what extent the components are synergistic and work together to positively influence PHIM and to potentially identify areas of misalignment to inform the design, implementation, use, and promotion of HIT used in PHIM. Particular attention will be paid throughout to describe the special needs of older adults.

## Methods

### Overview

We used a modified e-Delphi method to iteratively solicit, organize, and structure consensus judgments and opinions describing PHIM work by a panel of experts who provide PHIM assistance to health care consumers. Our modified Delphi method incorporated anonymity (ie, participants and their individual responses) and controlled feedback across multiple iterations of open-ended questions using Qualtrics web-based survey software. E-Delphi procedures have been used in health care [38,39] and health information management contexts [40].

### Ethics Approval

This study was approved by the University of Washington Human Subjects Division (STUDY00000768). Data were collected between April and December 2017.

### Recruitment

We recruited professional organizers with experience assisting health care consumers with PHIM to serve as key informants for this study. Key informants are often used because of their subject matter expertise and position in the community of interest, both of which give them deeper insight into the phenomenon of interest [41,42]. Professional organizers bring their talent to arrange artifacts and information. Furthermore, their experience in working with people facing situational and psychological challenges makes them astute observers of human behavior and effective communicators. Accustomed to aiding consumers, professional organizers have honed their ability to communicate intelligibly and comprehensively about the subject. In addition, professional organizers have a degree of objectivity and impartiality, given that they are removed from the artifacts and information they organize, as well as the people they assist.

Professional organizers were recruited in cooperation with leadership from the National Association of Productivity and Organizing (NAPO) professionals. Members of the NAPO with at least 2 years of experience as a professional organizer and experience organizing health information for at least 2 health care consumers in the last 4 years were eligible to participate. An evaluation of systematic reviews of Delphi studies in health sciences [43] found that the average number of participants in Delphi studies was in the lower to middouble digits. In our study, 23 members were eligible for the study and 16 participated in the Delphi process. Of 16 participants, 13 had 6 to ≥20 years of organizing experience and 6 to ≥30 PHIM health care consumers.

NAPO leadership announced the study via association events (such as webinars), their website, committees (eg, research and education), and special interest groups (SIGs; eg, Working with Seniors SIG and Technology SIG). The announcement included the study team contact information for questions and a link to a volunteer Qualtrics screening form with participant qualification questions.

### Data Collection

Essential to the Delphi method are the features of iteration and controlled feedback [44]. Controlled feedback was achieved by returning a cumulative summary of the results after each round, with opportunities for feedback on each question’s summary of responses. Participants augmented summary statements (often in the form of examples or additional details) and validated each question’s results summaries. After round 3, a final cumulative results summary (including summaries for all open-ended questions and charts summarizing responses to rank-ordered questions) was shared with the participants.

Multiple rounds with feedback opportunities allowed differing positions and consensus to emerge. Differing positions were documented in the results summaries and reported in the study findings. Consensus, defined as receiving no new information or insights, was reached by the end of 3 iterative rounds. An evaluation of systematic reviews of Delphi studies in the health sciences revealed that 2 to 3 rounds are typical for reaching a specified level of consensus [43].

Table 1 details the Delphi questions associated with each round. Each round provided an opportunity to ask follow-up questions to the prior round to further enhance cumulative understanding. The number of participants that responded to each question by round is also provided.

**Table 1:** Delphi questions by round and response type.

Delphi question	Round 1			Round 2		Round 3	
	Response type	Count^a^, n		Response type	Count, n	Response type	Count, n
1. How do you decide client needs, particularly when it comes to organizing health information?	I^b^		16	F^c^	10	F	6
2. What types of challenges do clients have in organizing their health information?	I		14	F	9	F	5
3. What barriers do you and other organizers face in helping clients organize their health information?	I		12	F	9	F	5
4. What are some noteworthy practices for organizing health information?	I		16	F	10	F	6
5. How does helping older adults (age 55 and over) organize and manage their health information differ from assisting younger adults?	N/A^d^		N/A	I	10	F	7
6. How is helping your clients organize health information different from organizing other types of information, or organizing in general?	N/A		N/A	I	10	F	6
7. What recommendations do you have regarding the best ways to communicate and discuss organizing health information as a service offered by organizers?	N/A		N/A	I	10	F	7
8. What do professional organizers need to assist clients with managing health information (eg, training, tools, etc)?	N/A		N/A	I	10	F	5
9. Please check your top 5 training or tool needs.	N/A		N/A	N/A	N/A	I	7
10. Please rank the following training needs by dragging and dropping each one, starting from most important (top) to least important (bottom).	N/A		N/A	N/A	N/A	I	7

^a^Count is the number of participants that responded to each question.

^b^I: initial response.

^c^F: feedback response.

^d^N/A: not applicable.

### Analysis

A total of 3 researchers with demonstrated experience and training in health informatics as well as behavioral, technical, and administrative information management analyzed the data. Throughout the analysis, the multiple perspectives of researchers, reflexivity (ie, constant internal scrutiny and questioning of researchers’ influence on the research process) [45], and the use of the constant comparative coding method [46] served as a check on researcher bias.

After each Delphi round, the first 2 authors summarized the participants’ responses [47] using open coding (eg, allowing codes to emerge from the data) and axial coding (eg, grouping related codes into categories). Themes were then identified among the categories to develop the Delphi summary report. The summary report was provided to participants at the end of each Delphi round for member checking.

Following participant validation, the first author used the Patient Work System model as a general schema to determine the fit of the identified themes to the model’s 4 components, including 3 context dimensions. The first 2 authors reviewed the coding results and agreed that a reasonable fit was achieved for the Patient Work System model to serve as an appropriate coding schema for research purposes.

The first author then used the Patient Work System model components as an a priori coding schema to recode the raw responses. Afterward, related codes within each component of the model were grouped into categories (eg, axial coding), and selective coding was used to identify the underlying themes and relationships among the model components (ie, derive key findings). Throughout the analysis, we referred to the PHIM literature [3] and literature that used and conceptualized the Patient Work System model [17,19,21,22]. This advanced the team’s understanding of the model constructs, the relationships among the model’s constructs, and our research data. The 3 researchers met every 1 to 2 weeks throughout the analysis process to review, discuss, and revise the coding as appropriate.

## Results

### Overview

As set forth, we used the Patient Work System model’s 4 components (person, tasks, tools, and context) to organize and describe the influences on PHIM work. Throughout our findings, special attention was given to influences related to older adults and interactions within and among the Patient Work System components. The key findings are summarized in Multimedia Appendix 1.

Representative participant quotations were used to illustrate the influence on PHIM work. As participants responded in incomplete sentences, text within brackets was used to clarify the meaning. In addition, the Delphi round and research question from which the quotation was drawn was noted in parentheses (R#Q#) at the end of each quotation to help clarify the meaning.

As noted in the introduction, the term *health care provider* is used throughout the paper to denote persons and places licensed to provide health care [4]. This term was selected because it was the most commonly used term by participants in referring to persons, such as physicians; nurses; physician assistants; therapists; social workers; those who provide dental, vision, and chiropractic care; and other “related” professionals and “peripheral” providers. For example:

[Individuals] also forget that peripheral providers such as dental and vision, chiropractic and other care providers also should be included in their health history.

R1Q2

To distinguish between health care provider persons and places, we append the word “organization” to health care provider in instances where participants’ responses indicate a place. Terms such as institution, clinic, hospital, rehabilitation, and pharmacy were variously used by participants in reference to places that generated PHI.

### Person

Our analysis revealed 2 key findings for the person component. The first key finding describes individuals’ PHIM goals. The second key finding describes how person-related factors converge to increase the PHIM workload and the level of capabilities needed to accomplish PHIM goals.

For the first key finding, we found that individuals undertook PHIM to achieve optimal health care outcomes, enable self-advocacy, and manage their health finances. According to key informants, individuals who sought assistance with PHIM wanted to ensure that their health care providers (1) had accurate and complete information, (2) did not misinterpret PHI received from other health care providers, and (3) were aware of any misdiagnoses or errors in their records. The following quotations describe health care and advocacy outcomes derived from managing medical PHI. The quotation about “optimal health care outcomes” was made within the context of avoiding conflicting and incomplete PHI that could lead to misinterpretation and misdiagnosis by their health care providers. Self-advocacy is mentioned in the context of an example of misdiagnosis correction:

Managing and sharing information is the goal for optimal health care outcomes.

R3Q5

Empowering [individuals] to be an integral part of their health by advocating for themselves [is a PHIM best practice].

R1Q4

The following quotation on managing health finances arose in an explanation of how situational disruptions in a person’s life forced a change in who handled finances in a household:

We see widows [or widowers, less often] who have never paid or analyzed bills or who have never been responsible for overseeing deductibles and out-of-pocket limits suddenly taking over where a spouse has been responsible.

R2Q5

For the second key finding, we found that individuals’ situational and psychological attributes converged in unique and dynamic ways to increase the PHIM workload and the capabilities required to do the work. As a result, individuals, or their representatives, sought assistance with PHIM. This key finding demonstrates the influence of interactions between situational and psychological attributes on the PHIM workload. The situational and psychological attributes derived from the data are presented in Table 2. In this table, situational attributes are organized by 4 factors (demographic, health status, life event, and capabilities), which describe the influences of an individual’s life circumstances on PHIM work. Table 2 also organizes psychological attributes into 4 factors (expectations, motivations, emotions, and attitudes). According to key informants, psychological factors signaled a person’s “psychological capacity to face (PHIM) tasks and the (personal health) information represented by the tasks” (R2Q2-3).

The dynamic interaction of situational and psychological attributes is illustrated by the following quotation. This quotation describes how a dramatic (ie, sudden or major) change in health status (ie, physical or mental) created a demand for new or more robust PHIM capabilities:

Sometimes a...[person’s] situation turns on a dime...[A person may have] routine issues with ongoing health information, but suddenly...[get] hit with a dramatic diagnosis, requiring research into [health care] options, multiple [health care] institutions, and [health care] providers. It was like a bomb went off.

R1Q3

**Table 2:** Attributes of individuals who sought personal health information management assistance by type and factor.

Factors		Attributes
**Situational**		
	Health status	Aging^a^, physical capabilities (hear, see, and move), and mental (memory, organizational, or ADHD^b^) issues
	Life events	Trauma, major illness, loss due to death, role shifts, relocation, downsizing, divorce, retirement, emergency
	Demographics	Age^a^, dwelling type, relocation, work status, independence, financial status, household or family structure, roles, support network, culture, and language
	Capabilities	PHI^a,c^, health finance^a^, information technology^a^, and health care and insurance (including associated terminologies)^a^
**Psychological**		
	Expectations	Goals, needs, priorities, preferences, demands, anticipation, required effort, PHI availability, and health future^a^
	Motivations	Willingness, interest, motivation^a^, time and energy^a^, desire^a^, stamina^a^, and tolerance for complexity
	Emotions	Confusion, fear^a^, shame, stress, overwhelm, stupidity^a^, worry, vulnerability, anxiety, and intimidation^a^
	Attitudes	Ambivalence, sensitive, trust of people (ie, privacy, confidentiality, respect)^d^, trust of information technology (ie, security)^a,d^, and personal safety^a^

^a^Attributes related to older adults (see the *Task*, *Tools*, and *Context* sections).

^b^ADHD: attention-deficit/hyperactivity disorder.

^c^PHI: personal health information.

^d^Person-level attributes related to PHI and technology (see the *Tools* section).

The responses indicated that interactions among situational changes related to aging (eg, health status, life events, and demographics) increased the demand for more robust capabilities (ie, knowledge, skills, and abilities). Key informants described how life events such as “downsizing, (moving into) assisted living or (a) nursing home...” may converge with demographic changes such as “becoming widowed/becoming a caregiver/losing a caregiver*.*” As a result, individuals may have to take on “new roles*.*” As the following quotation explains, older adults require additional health finance and health insurance capabilities to meet their newly acquired responsibilities:

[Older adults] may be more overwhelmed because they are less likely to have experience with financial responsibility. We see widows [or widowers, less often] who have never paid or analyzed bills or who have never been responsible for overseeing deductibles and out-of-pocket limits—suddenly taking over where a spouse has been responsible.

R2Q4

Psychological reactions to fluctuating situations also sometimes resulted in new capability demands or revealed capability deficits, particularly for older adults. Participants emphasized that the increasing volume and complexity of PHI from Medicare left some older adults feeling “intimidated or stupid” at a time in their life when they “know their medical history” is important. In addition, some older adults were less interested in taking on new PHIM responsibilities (motivations), spending time on PHIM (expectations), or learning new PHIM skills and technologies (health status and motivation), according to participants. The following response provides an example:

As a summary, the financial and medical lives of [older adults] are more complex [due to a greater likelihood of age-related health conditions] at the same time that their capacity for [or interest in] learning new techniques for managing information is waning.

R3Q5

### Tasks

#### Overview

A key finding for the tasks component outlined 5 PHIM tasks (ie, acquire, organize, process, reconcile, and store) that reflect the general workflow of an individual PHI document. Textbox 1 provides an abbreviated list of major PHIM activities undertaken for each task. A more detailed description of PHIM activities by task is provided in Multimedia Appendix 2. The following paragraphs provide a description of each task, associated key findings, and interactions with other Patient Work System components, where applicable.

**Personal health information management tasks and major activities described.**

**Acquire**

- Locate, determine how to access, and acquire personal health information (PHI) from patient portals and paper medical record repositories
- Enable multiperson authorization to acquire, if desired
- Document PHI sources, authorized individuals, portal user identification and passwords, and medical record requests

**Organize**

- Use a 3-part taxonomy (ie, medical, financial, and reference) to sort and house PHI (a standard adopted by key informants operating as professional organizers)
- Design and set up a personalized filing schema (a standard adopted by key informants operating as professional organizers) for each PHI classification considering usage priorities, transportability needs, modality (digital vs paper) preferences, placement requirements, schema familiarity, and complexity tolerance
- Determine and honor individuals’ privacy and confidentiality preferences for PHI (a standard adopted by key informants operating as professional organizers)
- Establish document control practices such as recording dates of service, page numbers, and marking as originals or copies
- Design tools (specific tools are described in the *Tools* section) using spreadsheets and paper forms to document, integrate, and track medical and financial PHI

**Process**

- Create and populate PHI tools (specific tools are described in the *Tools* section) to document, integrate, track, and share medical and financial PHI
- Maintain personal health information management system including acquiring new PHI and updating tools on a regular basis while preserving the 3-part taxonomy, personalized filing schema, and privacy and confidentiality preferences

**Reconcile**

- Understand discrepancies (such as inconsistencies and errors) in medical PHI, take steps to correct, document steps taken, and track to resolution
- Understand discrepancies in financial PHI by matching services provided with insurance coverage and payments made, file claim disputes, and document and track disputes to resolution

**Store**

- File PHI during and after completion of each task using personalized filing schemas according to disposition (ie, active, transportable, archival, discard, and backup)
- Abide by privacy and confidentiality preferences for all dispositions

First, it is important to note that although the PHIM tasks were presented linearly, the responses indicated that the tasks were carried out more dynamically in daily practice. Nevertheless, the sequence of tasks provided a general sense of the workflow for an individual electronic or paper document. The responses indicated that the acquisition and organization tasks were preparatory to the process and reconcile tasks.

The acquire task focused on gathering the required PHI. The organized task focused on sorting and filing for easy retrieval. The process task focused on extracting and entering PHI into formats to support sharing key elements of PHI with health care providers and caregivers and using PHI for self-care and handling personal finances. The reconcile task focused on resolving discrepancies in medical and financial PHI uncovered during the process task.

The store task varied based on the disposition of a particular document at a particular point in the workflow. That is, the storage location of a document depended on the timing, frequency, and place of access and on the type of PHIM activity being undertaken.

#### Acquire

Key informants described the acquire task as locating, accessing, and acquiring PHI from multiple sources, including retrieving PHI from patient portals and making medical record requests. Participants emphasized the importance of documenting the sources of PHI, acquisition procedures (instructions, usernames, and passwords), and the identity of individuals authorized to acquire PHI by the type of PHI they were authorized to acquire. Responses indicated that such documentation was seldom done by those who sought PHIM assistance but was the best practice of participants.

Interactions with the acquire task focused on the person, tools, and social context components related to authorizing personal stakeholders, including key informants, to acquire individuals’ PHI (particularly older adults). Responses indicated that authorization for multiperson access was needed for individuals unable or unwilling to acquire their own PHI. Moreover, older adults were more likely to (1) need assistance from others in acquiring their PHI, (2) not know that PHI was available in portals, and (3) forget portal passwords. Older adults were also less likely to know how to access patient portals or make medical record requests. In addition, some older adults feared offending health care providers when requesting copies of their PHI according to the participants.

#### Organize

The organize task appeared to be a design activity, or planning phase, and was described by participants as “creating a system to house information” (R2Q8). Overall, 2 activities for the organizing task were identified as key findings because of their apparent standing as PHIM practice standards for participants, that is, taken-for-granted principles for organizing PHI.

One key finding related to the organize task was that a 3-part taxonomy (medical, financial, and reference) was used to sort the PHI. The following quotation explains the importance of separating reference information from medical information:

[Individuals] attend support groups where they are given literature, they print things off the internet, and they clip articles from magazines; yielding piles of medically-related advice that may or may not be accurate, and it’s important that they don’t mix generalized information with their personal medical information.

R3Q4

Notably, participants paid little attention to the reference classification, beyond emphasizing the need to separate this type of information from medical and financial information.

The second key finding focused on personalizing the organizing schemas to the individual. The following response demonstrates the participants’ consensus that the possible schemas are numerous but what “works best” for the individual was paramount:

There are so many different ways to organize the same information—you need to have a discussion with the [person] to find out what way will work best for them. Some like to organize information by doctor, others by condition, and still others by year.

R2Q6

The participants indicated that personalized schemas were designed for each PHI classification. Responses almost exclusively described schemas for medical and financial classifications.

The primary interactions for the organize task were between the person and tools components. These interactions were apparent in the participants’ descriptions of the 6 parameters used by key informants in designing personal filing schemas:

1. Usage priorities (eg, a person’s immediate health issues, claim disputes, exercise routines, etc)
2. Transportability requirements (eg, take to a health visit, travel, emergency evacuation, etc vs paying bills, future tax filing)
3. Modality preference (person’s preference for digital, paper, or a combination)
4. Placement requirements inside and outside of the home to insure security, confidentiality, accessibility
5. Schema familiarity (eg, schemas currently used by individuals, such as reverse chronological order by health care provider specialty or name vs disease or problem)
6. Complexity tolerance (eg, a person’s medical situation or level of detail the person wants or can handle)

#### Process

Key informants defined the process task as “creating a system to process information” (R2Q8). The key finding for this task was that the work of the process task was extracting, entering, and tracking medical and financial PHI on an ongoing basis to support self-care, share PHI with health care providers, and monitor health finances. The following quotation speaks to dual medical and financial PHI focus:

The two biggest issues I see are:

1. Managing the financial component [of PHI] ([determining] what is covered? what has been paid/what [is] owe(d)? Is it the best plan as medical needs change?)

2. Managing the medical component [of PHI] (remembering appointments, tracking [the] taking [of] daily meds).

R2Q2-3

After the PHIM organizational structure was initially set up using the 3-part taxonomy and personalized filing schemas, medical and financial PHI was acquired, organized, and processed on an ongoing basis. Existing tools were used, and new tools were developed to help with the processing of PHI. The specific tools are discussed in the next section (see the *Tools* section). Some of the activities described by professional organizers in the process task include extracting, matching, and entering PHI using these tools. Monitoring and tracking PHI to ensure completeness and accuracy were also activities described by the participants.

Responses indicated that individuals shared processed PHI with their health care providers, family members, or others involved in their care. In addition, processed PHI was used to keep appointments, contact health care providers and insurers, keep track of self-care, and pay bills or ask questions about medical bills and insurance payments.

Interactions for the process task were primarily between the person and tools components. As exemplified in the *Person* section, the personal attributes of individuals influenced their ability to carry out process task activities. Participants explained that some individuals were unwilling or unable to commit the time and effort required for PHIM system maintenance. Alternatively, some individuals did not possess, and were unwilling or unable to acquire, the capabilities and motivation needed to continually process their PHI. According to the participants, older adults and those with complex health situations or organizing disorders, such as attention-deficit/hyperactivity disorder, were more likely to struggle with using PHIM tools and maintaining PHIM systems once implemented.

#### Reconcile

The fourth PHIM task, reconcile, focused on resolving the financial and medical PHI discrepancies identified in the process task. The key finding for the reconcile task revealed that reconciling medical billing and insurance payment discrepancies required financial proficiency, whereas correcting discrepancies in medical PHI remained elusive.

In resolving financial discrepancies, professional organizers outlined a step-by-step process. First, an understanding of the medical billing process and insurance coverage was required. Specifically, individuals needed to understand (1) whether the service provided was covered by insurance, (2) who was responsible for submitting bills to insurance (patient or health care provider), and (3) who was to be reimbursed (patient or health care provider). Second, individuals were required to determine whether the copays, coinsurance, and deductibles had been satisfied. Third, individuals needed to decide whether a claim should be filed, a charge should be disputed, or a denial should be appealed. If yes, the process for filing a claim or disputing a charge or insurance denial was investigated. Fourth, documents were submitted to file the claim or dispute the charge or insurance denial. Fifth, each claim or dispute was tracked to resolution.

According to the participants, this process required a level of proficiency that many individuals seeking PHIM assistance lacked. The responses indicated that some professional organizers may also lack the necessary financial proficiency. The following quotation was given in response to the Delphi question about the training and tools needed for organizers to provide PHIM assistance:

I would hope that “professional” organizers would recognize when they are in over their heads. Not all professional organizers are qualified to be assisting with medical and financial information. Maybe this is better suited as a service offered by Professional Money Managers?

R2Q8

Regarding the reconciliation of discrepancies in medical PHI, the pathway to correction was unclear to professional organizers. Responses included, “It’s nearly impossible to undo the misdiagnosis or interpretation*”* (R3Q4); “patient portals provide no method by which the information can be reported/corrected*”* (R3Q1); and “when you attempt to electronically share or update information, it isn’t done*”* (R2Q6).

Interactions for the reconcile task focused on the person-task interactions of older adults. Specifically, responses indicated that older adults had difficulty understanding and carrying out reconcile activities. According to the participants, the financial situation and insurance mix of the older adults were more complex. In addition, their health issues were worse, and health care providers were more numerous. Consequently, there was more medical and financial information to manage and more inconsistencies to resolve. Moreover, older adults’ personal situations were more likely to undergo change because of aging. For example, older adults may be required to assume new PHIM roles, such as managing household finances, at a time in life when they are unwilling or unable to learn the required skills or devote the required time and effort.

#### Store

The fifth and final task, store, entailed storing PHI according to disposition, as other PHIM tasks were carried out and completed. The key finding for the store task was that 5 storage dispositions (active, transportable, archive, discard, and backup) were used to file documents based on the timing, frequency, and location of access and stage in the PHIM document workflow.

Responses indicated that active disposition was used for the PHI that was needed at hand to complete PHIM processing tasks and support daily care. Examples included holding a medical bill in an active file until payment is made by insurance and holding a visit summary for data extraction. PHI was also kept at hand to support daily care (such as taking medications, exercising, following diet instructions, and updating monitoring logs). The transportable disposition was used for PHI taken to health appointments to share with health care providers, carried on individuals for emergencies, and taken during disaster evacuations or other travel. The archival disposition was used for PHI needed in the future for taxes, end of life, and passing on to future generations. The fourth disposition, discard, was reserved for unnecessary duplicates and PHI that had been scanned or otherwise was no longer needed. PHI designated for disposal was shredded and discarded. The fifth disposition, backup, was used as a second copy of the PHI stored in a separate location from the original, whether digital or paper.

Primary component interactions for the store task were found between the task and person components related to the discard disposition, that is, the responses indicated that discarding PHI was the most difficult of the storage dispositions for individuals. Key informants asserted that individuals did not know what PHI to keep, toss, or shred “in case they might need it someday” (R1Q3). Furthermore, professional organizers ranked the need for guidelines similar to those provided for the disposal of financial records as a top priority for PHIM work. The following quotation explains this:

I have many years of healthcare administration experience, which gives me some insight into paperwork. Yet, I don’t have a codified list to turn to when deciding what is important enough to keep and what can be shredded. For financial documents, I refer to IRS publications and commonly held accounting principles. There is nothing similar for healthcare documents.

R2Q8

### Tools

The key finding for the tools component was that integration tools were used heavily to (1) provide a comprehensive summary of an individual’s medical history and self-care activities, (2) match medical expenses with insurance claims and payments, and (3) reconcile discrepancies in medical and financial information. Textbox 2 provides a list of PHIM integration tools and technologies. A complete list of the tools and technologies identified by professional organizers is provided in Multimedia Appendix 3. The following paragraphs focus on the integration tools used specifically for the process and reconcile tasks, which were emphasized in our data as comprising the bulk of PHIM work.

**Figure 2:**
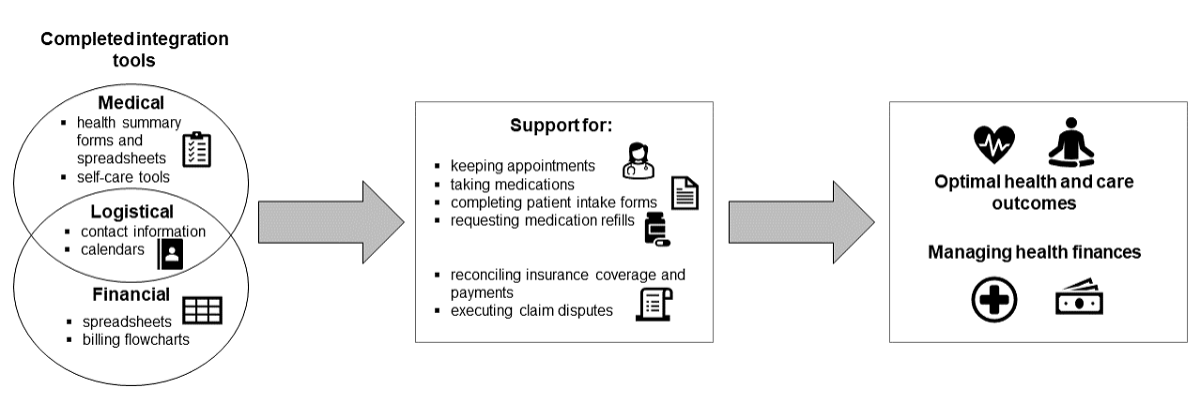
Integration tools used and their impact.

As depicted in Figure 2 and Textbox 2, key informants described 3 categories of tools used to integrate PHI into the process task: medical, logistical, and finance. Representative quotations identifying these integration tools are provided in Multimedia Appendix 4.

Primary interactions were found between the tools and person components, as evidenced by the personalization of the PHIM organizational system to the capabilities of the person, particularly older adults. Although not restricted to older adults, participants emphasized that health complexity coupled with limited PHIM and technology capabilities were more common among older adults who sought PHIM assistance. The following response demonstrates the need to tailor the PHIM system to fit the capabilities and health needs of subgroups of older adults:

The needs of someone 55 [years old] are much different [from someone of] 70 and much different from [someone of] 90. In my family, we have one person in each age group, and it is amazing how different the [PHIM] systems are that are needed within the category of older adults simply based on age.

R3Q1

**Integration tools and technologies used for personal health information management work across and within tasks.**

**Crosscutting**

- Computer devices (desktop, laptop, tablet, and phone including type [ie, Macintosh or PC]), software, and internet connectivity were used in multiple tasks including acquire, process, reconcile, and store

**Acquire**

- Portal access documentation including instructions and authorizations for who can access personal health information (PHI) and what PHI they can access
- Forms to authorize and document stakeholder access to PHI
- Digital devices and software used to download and transfer PHI

**Organize**

- PHI 3-part organizing taxonomy (medical-financial-reference)
- Personalized filing schema for each classification of PHI considering usage priorities, transportability needs, modality preferences, placement requirements, schema familiarity, and complexity tolerance

**Process**

- Medical
  Health summary forms and spreadsheets used to summarize active medical issues and problems, family and personal medical histories, medications and supplements, and medical encounters and visits
  Self-care tools including logs and journals, treatment information, reference documents, medication, and supplement materials (eg, administration and refill schedule, compliance log, and pillboxes)
- Logistical (health care provider and insurance)
  Business cards, schedulers, calendars, spreadsheets, contact software, and address books
- Finance
  Spreadsheets used to match medical services and charges with insurance coverage, bills, explanation of insurance benefit statements (ie, explanation of benefits), and payments
  Billing flowcharts by health care provider

**Reconcile**

- Spreadsheets to document and track to resolution claim disputes and requested medical record changes

**Store**

- Digital devices (cloud, thumb and hard drives, CD, and DVDs) and software (Box, Evernote, Dropbox, Google, etc)
- File containers, binders, folders, dividers, business card organizer sleeves, and sheet protectors

As described in the *Task* section, the tolerance for complexity was a parameter in designing the filing schema. In addition to health complexity and capabilities, participants observed individuals’ current practices before determining the overall PHIM system design. The following response provides an example:

If one is used to dumping paper all over, a simplified paper system may be acceptable; labor-intensive scanning and digital systems probably will not be.

R3Q6

In general, opting for a simpler PHIM system that draws on the familiar was best for older adults according to the participants. Though older adults “have learned they need to know their medical history” (R2Q5), according to professional organizers, older adults were less likely to want to learn new things and be involved in PHIM work. Moreover, participants pointed out that older adults were more likely to have physical and mental limitations (eg, physical, cognitive, mobility, hearing, and visual limitations) that negatively influenced their ability to perform PHIM work:

For older [individuals], the system needs to be streamlined and simplified. It needs to follow other patterns they have used for organizing data. Staying with a known system and adapting it to medical information makes it easier for the older [adult] to use.

R2Q5

Participants also noted that older adults were more likely to want paper copies of their laboratory test results and other health reports. Thus, the participants argued that older adults may be more likely to use a paper-based organizing system.

### Context

#### Overview

Similar to Holden et al [19,21], we used 3 dimensions (organizational, social, and physical) to present our findings regarding contextual influences on PHIM work. Overall, 2 key findings for the organizational context and one each for the social and physical contexts were revealed during the analysis.

Key findings for the organizational dimension indicated that the attributes of insurer-generated and health care provider-generated PHI and repositories increased the PHIM workload. The key finding for the social dimension indicated that the involvement of personal stakeholders (family, friends, caregivers, and helpers, including PHIM assistance providers) assisted with the PHIM workload but created sensitive interpersonal dynamics and complicated maintaining information controls. For the physical dimension, the protection of PHI held in the physical and acquired digital spaces of individuals who sought PHIM assistance was important but inconclusive.

#### Organizational Context

**Figure 3:**
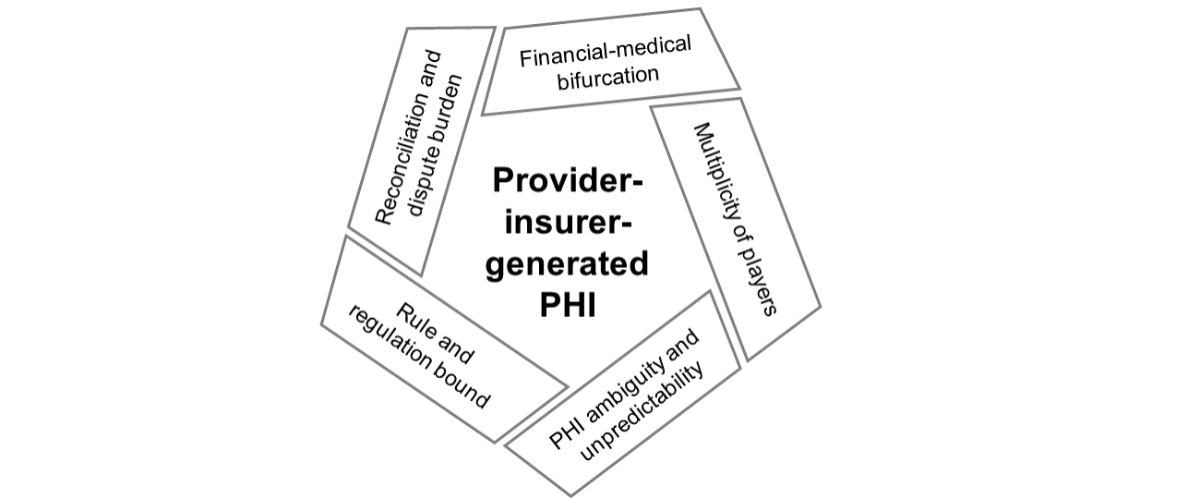
Distinctive attributes of provider and insurer generated personal health information (PHI).

The first key finding for the organizational context was that attributes of health care provider- and insurer-generated PHI increased PHIM workload. These attributes included (1) financial-medical PHI bifurcation, (2) multiplicity of players, (3) PHI ambiguity and unpredictability, (4) rule and regulation bound, and (5) reconciliation and dispute burden (Figure 3). The subsequent section provides a description of each attribute and its influences on PHIM tasks. Representative quotations are provided in Multimedia Appendix 5.

The first attribute, bifurcated PHI, resulted from PHI generated from 2 separate sectors of health care (ie, providers and insurers) for a single episode of care or service. According to key informants, in other types of personal information management work, a single document was generated by a single source describing the service provided and the cost of the service (eg, a utility bill). The second attribute, a multiplicity of players within each sector, generated PHI documents for a single episode of service (eg, separate facility and professional bills; Medicare and private insurer statements). According to the participants, the volume and diverse sources of PHI created greater task complexity compared with other types of information management tasks.

A third attribute, the ambiguity and unpredictability of PHI generated by health care providers and insurers, made it difficult to track PHI and schedule PHIM work. According to the professional organizers, each player within the sectors had their own PHI release or distribution cycle. Moreover, participants explained that communications within and between the health care and insurance sectors were uncoordinated and unclear (eg, lack of uniformity in terminology and format). Furthermore, the lack of transparency in policies and procedures and the different terminologies used to describe the same thing made it difficult to identify and resolve discrepancies.

Fourth, the rules and regulations bound attribute of health care provider-generated and insurer-generated PHI-guided and constrained PHIM work. For example, according to the participants, not understanding which services were covered by insurance or how to dispute an unpaid claim could have financial and health consequences. Fifth, the participants indicated that the burden of regularly reconciling and disputing medical and financial PHI discrepancies was unique to PHIM. Other types of home finance management did not require such detailed surveillance.

The second key finding for the organizational context was that multiple, imperfectly connected, and constantly changing health care provider and insurer PHI repositories required constant surveillance, downloading, and updating to keep the PHIM system current. Representative quotations are provided in Multimedia Appendix 6. Key informants agreed that maintaining the PHIM system once it was established was one of the biggest challenges for individuals.

#### Social Context

The key finding for the social context was that the involvement of personal stakeholders assisted with the PHIM workload but created sensitive interpersonal dynamics and made maintaining information access controls complicated. In general, responses indicated that interpersonal dynamics were more sensitive, and information controls were more complicated for older adults. We found that the influence of personal stakeholder involvement on interpersonal dynamics and maintaining information controls was primarily derived from interactions with the person and tasks components of the work system model. Representative quotations are provided in Multimedia Appendices 7 and 8.

The responses indicated that PHI was highly charged for some individuals. Professional organizers described the shame, embarrassment, and discomfort that some individuals felt when sharing or discussing their PHI. In addition, responses indicated that when a personal stakeholder suggested that assistance with PHIM work was needed, some individuals avoided or resisted help.

Medication management was an area of stakeholder involvement that created sensitive interpersonal dynamics with key informants in their role as professional organizers. Responses indicated agreement among participants that managing medication information was a part of PHIM work (ie, tracking dosages to take and when, scheduling refills, and organizing pill bottles). However, some key professional organizers cautioned against filling pillboxes because of potential liability. Instead, they suggested recruiting another personal stakeholder to help with this activity.

Maintaining information controls was also complicated by the interactions between the social context and individual components. Person attributes complicated maintaining information controls because one person filled multiple roles, and multiple people were involved in one or more people’s PHIM. For example, some individuals filled multiple care roles (eg, self-carer, care-receiver, caregiver, and advocate), which necessitated access to their PHI by others and access to others’ PHI.

In addition, some individuals needed assistance to complete multiple PHIM tasks, revealing interactions between the social context and specific tasks. As professional organizers, participants needed access to the person’s PHI to design and set up a PHIM system. In addition, the responses indicated that multiple personal stakeholders could be involved in maintaining the PHIM system and advocating for the individual in reconciling medical and financial PHI discrepancies. The more people involved in PHIM, the more complicated managing information controls became.

Responses indicated that older adults were more likely to serve dual roles as caregivers and care receivers (ie, be in a generational sandwich) and face “intergenerational struggles over who was in charge*.*” Hence, managing information controls and interpersonal dynamics were more complex for older adults.

#### Physical Context

For physical context, the key finding was that health care consumers’ PHI be protected from hazards and remain accessible only to authorized individuals. However, the security of PHI housed in the diverse physical and acquired digital spaces of individuals remained inconclusive. Primary interactions among the Patient Work System components elucidate this key finding, as described subsequently. Representative quotations are provided in Multimedia Appendix 9.

PHI protection was characterized by interactions between 2 context dimensions (physical and social) and the person component. The person component clarified individuals’ privacy expectations about PHI held in their physical and digital spaces (ie, physical context) and revealed several parameters related to personal stakeholders’ access (ie, social context). To gain trust and honor individuals’ privacy expectations, professional organizers said that they promised to maintain individuals' confidentiality and protect their PHI. In addition, participants pointed out that some individuals wished to keep only certain PHI “hidden” (eg, certain health conditions or medications). Notably, the responses did not indicate how the necessary information controls to achieve these requirements were to be achieved.

Further complicating PHI protection, responses showed that the physical context of PHI protection was situated inside and outside of the home in physical environments and digital spaces (eg, devices and storage spaces) acquired by individuals. In response to the diverse physical contexts requiring PHI protection, professional organizers indicated that each personal stakeholder’s access rights should be clarified and documented to include the following: (1) specific PHI authorized for access, (2) tasks authorized to carry out, (3) mode of access, and (4) location of access. The responses did not specify how these parameters were put into practice.

The complexity of maintaining information controls given the multiple PHIM roles and tasks taken on by individuals and their personal stakeholders was described in the *Social Context* section. Protecting individual’s PHI in and outside the home (ie, physical context) and stored in digital and paper modes (ie, tools component) while hiding some of their PHI from specific stakeholders (ie, *Social Context*) further added to the complexity of maintaining the information controls described in the *Social Context* section. Although the responses clarified these complications, the measures taken to control access to PHI individuals’ wished to remain hidden were not delineated.

Interactions between the physical context and the tools component suggested uncertainty among professional organizers concerning the selection and use of secure digital tools. For example, key informants named a number of digital tools that could be used to store PHI, including Evernote, One Note, Box, Dropbox, Google, and on the cloud. Responses indicated that participants’ understanding of the ability of these tools to protect individuals’ PHI from unauthorized access varied. For instance, several responses vaguely asserted that “specific sites/apps that keep health records securely in the cloud” (R1Q4) could be used to allow remote access. One response indicated that Evernote was not Health Insurance Portability and Accountability Act (HIPAA) compliant but that note-level encryption could be used. Another response indicated that Box was HIPAA compliant and more secure than Dropbox. Further indicating uncertainty around the best tools to use to ensure PHI protection, key informants ranked HIPAA guidelines for health care consumers as one of their top 5 PHIM tool needs.

Interactions between the physical context dimension and the tools component revealed 4 PHI protection best practices. Only one of these practices was mentioned in relation to digital PHI, further suggesting that the protection of digital PHI, in particular, was uncertain. One practice was to create a backup copy of PHI (digital and paper) and store it in a separate location from the original. A second practice was keeping paper PHI away from environmental hazards (eg, animals, small children, plumbing, windows, and fans). Third, professional organizers asserted that the use of visual cues (eg, colored file folders for paper PHI and placing notes on refrigerators or mirrors) made finding and using information easier. For PHI, participants recommended modifying or not using visual cues to avoid making it susceptible to privacy and security breaches. The fourth practice was shredding PHI that contained confidential information when no longer needed.

## Discussion

### Principal Findings

#### Overview

**Figure 4:**
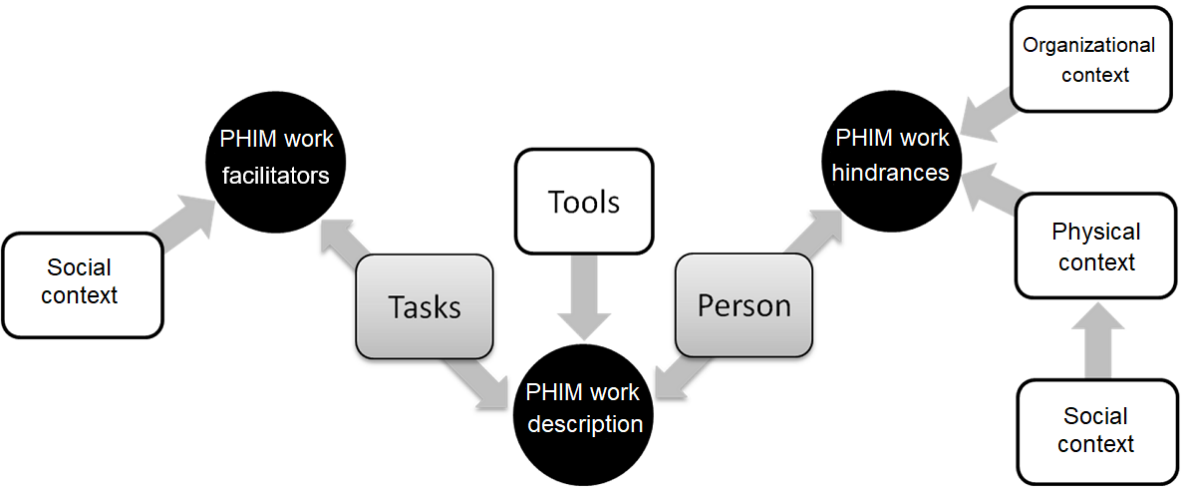
Contributions of components of the Patient Work System model to study objectives. PHIM: personal health information management.

Our key findings further specify the Patient Work System model, provide a detailed description of PHIM work, and delineate the influences that facilitate and hinder PHIM work. As shown in Figure 4, key findings describing PHIM work were derived from the person, tasks, and tools components of the Patient Work System model. Influences that facilitated and hindered the PHIM work of health consumers were derived from interactions among the 3 context dimensions and the person and tasks components. The involvement of personal stakeholders (social context) eased the task burden of health care consumers (person component) but complicated the management of PHI controls (physical context). A list of key findings organized by study objectives and Patient Work System components is provided in Multimedia Appendix 10.

#### PHIM Work Description

The person component revealed individuals’ PHIM goals, namely, achieving optimal health and care outcomes, enabling self-advocacy, and managing health finances. For the task component, we found that a 3-part PHI organizing taxonomy (ie, medical, financial, and reference) was used to sort PHI. We found that PHI documents flowed through 5 PHIM tasks (ie, acquire, organize, process, reconcile, and store) that were identified. Finally, for the task component, 5 storage dispositions were identified: active, transportable, archive, discard, and backup. Arguably, the most important finding was from the tools component, which crystallized the function of integration and reconciliation tools. These tools were essential in creating comprehensive and accurate health and finance PHI summaries to support self-care, share with health care providers, and work with insurers to ensure financial fidelity.

#### Facilitators

Regarding interactions between the person component and social context, we found that personal stakeholders facilitated consumers’ PHIM work. They supplemented the required competencies, assumed part of the workload, and designed the needed integration and reconciliation tools. In addition to professional organizers, personal stakeholders volunteered, asserted their role through the person receiving assistance, or were recruited by the health consumer or professional organizers.

In particular, professional organizers (social context) contributed to the PHIM work of individuals by personalizing the filing schema for each classification of the organizing taxonomy (task component). Findings suggest that personalization increased the likelihood that health care consumers would use and maintain the PHIM system. In addition, tailoring the schema to individual needs and preferences eased the workload burden and competency requirements of health care consumers.

Filing schemas were personalized using 6 parameters (ie, PHI usage priorities, transportability needs, modality [digital vs paper] preferences, placement requirements, schema familiarity, and complexity tolerance). For example, we found that creating a current and past health summary using health care provider-generated PHI and tracking medical bills and insurance claims using health care provider-generated and insurer-generated PHI were 2 PHI use priorities for health care consumers. Consumers’ preference for a paper or digital tool, the filing schemas currently being used by consumers (eg, organizing by health care provider name, specialty, and date of service), where the specific PHI will be used (clinic visit, emergency, and evacuation), and the level of complexity the consumer can handle were filing schema considerations.

#### Hindrances

Personal stakeholders’ involvement (social context) also hindered PHIM work. Stakeholder involvement created sensitive interpersonal dynamics (person component) and complicated information control management (physical context). Our findings indicate that personal stakeholders filled multiple roles and tasks situated inside and outside of individuals’ homes. Moreover, PHIM was a highly charged subject for health care consumers, which resulted in nuanced privacy expectations that further complicated maintaining information controls.

From the physical context dimension, we found that health care consumers housed the PHI they obtained and generated in their physical environments (ie, homes, cars, and wallets) and acquired digital spaces (ie, digital devices, software, and cloud storage). Health care consumers (personal component) and professional organizers (social context) recognized that privately held PHI should be protected from environmental hazards and unauthorized access. Despite their recognition, our findings indicated that health care consumers and professional organizers lacked clear solutions for maintaining appropriate information controls and securing privately held PHI, especially digital PHI.

**Figure 5:**
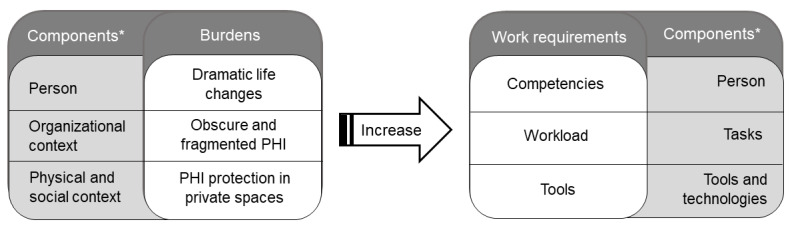
Personal health information management burdens arising in the person component and dimensions of the context component increase health consumer work requirements in the person, task, and tools components. *Patient Work System model components (person, tasks, tools and technologies, and context) and dimensions (organizational, social, and physical) of the context component. PHI: personal health information.

As depicted in Figure 5, interactions among influences in the person component and the 3 dimensions of the context component increased the PHIM work requirements. The number and sophistication of the competencies needed to complete tasks and use tools increased with dramatic (ie, sudden or major) life changes (ie, person component). For instance, we found that dramatic changes in health status (ie, physical or mental) or personal circumstances (death of a spouse, moving into assisted living, or making Medicare and Social Security decisions) simultaneously precipitated the need for a deeper understanding of an individual’s PHI and increased its complexity. Workload (tasks component) expanded with the increased complexity of PHI (person component), as did the need to integrate and reconcile the growing volume of PHI that was obscure and fragmented (organizational context).

Furthermore, our findings suggest that health care consumers’ PHIM support (social context) and training needs (person component) and the fragmentation of PHI generated by health care providers and insurers (organizational context) operate together to increase the PHIM workload, required competencies, and sophistication of the tools needed at a time when individuals’ capacity to take on the added workload or develop new PHIM competencies is waning. This is especially true for older adults and those with multiple chronic conditions but is likely applies to anyone with a debilitating condition.

### Comparison With Prior Work

Our description of PHIM work contributes to a growing body of work that relies on the Patient Work System model and its permutations [5,16-18,27] as a framework for understanding patient work [19,21,22], including PHIM [23-26].

Consistent with the existing literature [3], our description of PHIM work relied heavily on the person, tasks, and tools components to portray a familiar array of person characteristics [9,23,24,39,48-50] and lists of analogous tasks [8,9,23,24,48,49,51] and tools [8,9,23,39,48,50,51] used by health care consumers. Our findings make unique contributions to PHIM descriptions, with the introduction of health finance management as a major PHIM goal for health care consumers. To date, research has been limited to health care consumers’ ability to understand and use insurance information effectively [52,53], their management of out-of-pocket expenses [11,24,54], and their desire to talk to their health care providers about health care costs [11,55].

In our search of web-based gray literature, we uncovered 2 disease-specific websites [56,57] and money management [58] and tax preparer [59] websites that offer guidance to health care consumers on tracking their medical bills and insurance claims. We also found web-based health care provider organizations [60] and consumers [61] websites that offer education to help consumers estimate their out-of-pocket health expenses. Our findings, along with these websites, indicate that health care consumers take a broader view of health finance management than is currently addressed in the research literature.

In their 2009 report, Agarwal and Khuntia [62] called for the articulation of health care consumers’ workflows and processes. To our knowledge, ours is the first study to outline the flow of PHI documents through PHIM tasks (ie, acquire, organize, process, reconcile, and store) and to provide detailed descriptions of the 2 processes (ie, integration and reconciliation) used by health care consumers that required the creation of PHI tools to achieve their PHIM goals. Furthermore, for the storage task, we add 2 storage dispositions (ie, discard and backup) to those already identified in the literature (ie, active or at hand [8,9,24,48], transportable [9,24], and archived [8,9,24]).

Finally, our description of PHIM work includes a 3-part organizing taxonomy (ie, medical, finance, and reference) that diverges from the 3-part taxonomies published in the literature. In our study, the medical classification included patient-generated health data [63,64], health care provider-generated data (referred to as clinical data in the biomedical literature [65,66]), and health care provider-generated data that were extracted and entered into forms created by health care consumers. Vincent et al [66] referred to this PHI as professionally sourced, rekeyed PHI. Our review of the literature found that rekeyed PHI was not recognized [65] or was excluded [66] from other 3-part taxonomies and policy makers’ patient-generated health data definitions [63,64].

In our study, the practice of extracting medical and financial PHI from clinical and insurance records and entering (or rekeying) them into summary forms is referred to as *integration work*. The integration of health care provider- and insurer-generated data has driven much of the PHIM work described in our study. We found some PHIM studies with older adults [9,24,50] and people with multiple chronic conditions [54] that also reported the integration of many of the same types of PHI, although not to the degree found in our study.

In recognition of PHI fragmentation, the Office of the National Coordinator for HIT has required select vendors [67] to develop secure, standards-based application programming interfaces (APIs) for patient portals. APIs may support apps that allow health care consumers to automate the integration of medical and financial PHI available through patient portals. However, early evidence on the benefits of API-leveraged apps for health care consumers is inconclusive. Johnson et al [68] found that downloading medical information from a portal significantly increased (17%-32%) from 2017 to 2020. Transmitting medical information from a portal to a health care provider (10%-17%) also significantly increased over this period, whereas transmitting medical information to a caregiver or health application remained stable (3%-5%).

### Strengths and Limitations

One of the strengths of this study is the use of professional organizers who provide PHIM assistance to health care consumers as key informants. Consistent with 3 criteria by Tremblay [42], professional organizers are experienced in the community of interest (ie, assisting health care consumers with PHIM) and the subject of interest (ie, experts in organizing PHI and other artifacts). Organizers are also skilled in communicating their knowledge and experiences intelligibly and with impartiality. As a result, we were able to use the person, tasks, and tools components to provide a rich, detailed description of PHIM work and characterize the personal and contextual influences that hindered and facilitated PHIM work—a second strength of the study.

A limitation of the study, however, was that health care consumers whom professional organizers assisted were not included in the study. The inclusion of health care consumers in future studies is needed to validate organizers’ perspectives.

A second limitation is that professional organizers were not randomly selected. Although we used purposive sampling to identify experts, all were volunteers from the same association. The conduct of additional studies with other professionals who provide PHIM assistance to health care consumers is needed to validate organizers’ perspectives and capture any divergent views. More research is needed that examines PHIM from a professional perspective. Professional organizers’ responses indicate that other professionals, such as money managers, may provide health care consumers with PHIM assistance. There is a need to understand the range of professionals involved in providing PHIM assistance, their perspectives on the PHIM work of health care consumers, and their approach to providing assistance. Additional research with professionals will enrich our understanding of the PHIM needs and practices of health care consumers, as well as the changes needed to relieve consumers’ PHIM burden.

Third, there may be members with the required experience who did not volunteer. We worked under the leadership of the association to ensure broad participation. However, volunteers may have different views.

Fourth, evidence suggests that health care consumers whose PHI was being managed were well resourced and insured. They were able to engage the services of professional organizers, and managing financial PHI (including insurance) was emphasized in PHIM work. Our approach purposefully removed economic constraints to better understand the challenges that remain around patient engagement [11,13] and PHIM work for older adults. Therefore, caution should be exercised when transferring our findings to uninsured and low-resource groups. Furthermore, further studies are needed to examine the applicability of our findings to health care consumers of diverse ages and economic circumstances.

Fifth, health care consumers who engaged professional organizers in assisting with PHIM may be a unique subgroup. Ancker et al [54] found that the efforts of health care consumers with multiple chronic conditions to correct errors in their medical and financial information resulted in lengthy PHIM projects. Similarly, health care consumers who engaged professional organizers to assist with PHIM may need assistance because of the complexity of their personal circumstances or health conditions.

### Future Directions

Our description of PHIM work reveals several opportunities for research and areas for policy and consumer health technology (CHT) development. Research is needed to understand and support health care consumers in acquiring and managing their financial PHI. Our findings indicate the pivotal role of health finance management for consumers. The limited research on this topic and calls for health care consumers to control health care costs [69] are further indicators of the need for more research. Further studies are needed to confirm or refine our description of the PHIM and PHI document workflow.

Existing efforts to aid consumers in maintaining active, transportable, and archived PHI files should include paper format. As our study found that health care consumers have a need to discard and back up their PHI, policy makers should consider developing PHI retention and backup guidelines for health care consumers. Tools (paper and digital) for securely storing, backing up, and safely discarding health care consumer–held PHI are also needed.

Hindrances that add to health care consumers’ PHIM burden or interfered with PHIM work need to be resolved. Our study suggests that rather than relieving health care consumers of the burden of sharing their PHI across providers, health care portals have increased individuals’ PHIM workload. Our findings indicate that the fragmented PHI generated by health care provider and insurer portals has resulted in health care consumers extracting medical and financial PHI from clinical and insurance records and entering (or rekeying) it into summary forms. These summary forms help them manage their health and finance and share their PHI with health care providers, insurers, and caregivers.

Research is needed to understand the nature and prevalence of PHI integration into the PHIM of health care consumers. In addition, we urge health care provider and insurer organizations work to reduce their contribution to the PHIM work burden by improving the consistency, clarity, and formatting of the PHI they generate.

CHT solutions are also required to eliminate the integration work of health care consumers. Specifically, we recommend that CHT be developed, which automates the creation of comprehensive current and past health summaries that can be provided to health care consumers in paper and digital formats. Similarly, for financial PHI, CHT is needed to track consumers’ medical bills and insurance claims, flag inconsistencies, and generate claim appeal forms. The tools created should allow easy extraction from patient records and automatic entry into a standard format that can be printed, faxed, and filed electronically.

### Conclusions

In this study, we examined 4 components (person, tasks, tools, and context) of the PHIM work system, paying particular attention to contextual dimensions and interactions among components. We also explored possible pertinent issues related to PHIM, as observed in older adults.

In summary, our findings support the assertion [11,70] that the conduct of PHIM work facilitates health care consumers’ management of their health, health care, and health finances. Importantly, we also found that health care consumers’ attributes converged with the organizational contexts within the health system to increase the PHIM workload for consumers. We found that the workload demand was even greater for older adults and those with multiple chronic health conditions or organizing disorders. Furthermore, we found that when the PHIM work burden exceeded the capacity of health care consumers, personal stakeholders were called upon, or voluntarily stepped in, to provide PHIM assistance. Although needed to relieve the workload burden, our findings suggest that the involvement of personal stakeholders added to PHIM work complexity, particularly around maintaining information controls for multiperson access [3,24].

At a theoretical level, our study contributes to PHIM literature in several ways. First, we specify the social, organizational, and physical contexts. We adopted a systemic approach and used professional organizers as experts to demarcate the definitions and dynamics of the PHIM system components and dimensions (description of the elements and explication of how they interact). This enabled us to address an area of PHIM research that has not been fully clarified, particularly regarding the definition of the context. Second, our analysis yielded detailed information on the interactions among the components of the PHIM system. Our analysis also uncovers the nature of PHIM workload or burden and—notably—points out the role of organizational factors in creating such a burden. Third, we add to the research investigating PHI (paying close attention to health care provider-generated, insurer-generated, and patient-generated PHI and definitions thereof) and PHIM tools (indicating the special role of integration tools).

At a practical level, the results of this study will be valuable to health care providers, policy makers, and system designers. Health care providers may use the insights presented here to remain sensitive to the issues and challenges patients face and offer assistance from an organizational standpoint. Policy makers may develop guidelines and policies concerning PHI protection or retention based on our findings. CHT designers can leverage our findings concerning the role of context in PHIM and the flow of PHI documents throughout PHIM tasks to propose features that would support PHIM or reduce the burden experienced by health care consumers. We also discussed 5 storage dispositions that CHT designers should consider when devising appropriate PHIM solutions.

We encourage further research in this area, particularly in investigating the complexity of PHIM. The burden experienced by patients, especially older adults; PHI definitions or categories; and the management of PHI require further attention.

## Appendix

Multimedia Appendix 1 Key findings by Patient Work System model component.

Multimedia Appendix 2 Description of personal health information management activities by task.

Multimedia Appendix 3 Description of the tools and technologies used in personal health information management work across and within tasks.

Multimedia Appendix 4 Representative quotations for lists of integration tools used in the process and reconcile task.

Multimedia Appendix 5 Representative quotations for the distinctive attributes of personal health information generated by insurers and providers.

Multimedia Appendix 6 Representative quotations for attributes of provider and insurer personal health information repositories.

Multimedia Appendix 7 Representative quotations that demonstrate how personal stakeholder involvement interacts with other components of the Patient Work System to create sensitive interpersonal dynamics.

Multimedia Appendix 8 Representative quotations that demonstrate how personal stakeholder involvement interacts with other Patient Work System components to complicate information control maintenance.

Multimedia Appendix 9 Representative quotations that demonstrate how the physical context interacts with other Patient Work System components to influence the protection of personal health information.

Multimedia Appendix 10 List of key findings by Patient Work System components and study objectives.

## Abbreviations

API: application programming interface
CHT: consumer health technology
HIPAA: Health Insurance Portability and Accountability Act
HIS: health information system
HIT: health IT
NAPO: National Association of Productivity and Organizing
PHI: personal health information
PHIM: personal health information management
SIG: special interest group

## Data Availability

In the multimedia appendices, we provide trace tables that include representative source data to support our findings. The full data sets generated during and analyzed during this study are available (to the extent possible under the institutional review board guidelines governing the study) from the corresponding author on reasonable request.
