# Supplementary material for: A Description of Personal Health Information Management Work With a Spotlight on the Practices of Older Adults: Qualitative e-Delphi Study With Professional Organizers
Source: J Med Internet Res. 2023 Mar 31;25:e42330. doi: 10.2196/42330 (PMC10131782; doi:10.2196/42330)
Supplement: Multimedia Appendix 1 [file jmir_v25i1e42330_app1.docx]

| Multimedia Appendix 1 Key findings by Patient Work System model component. | |
| --- | --- |
| Component | Key finding |
|  |  |
| **Person** |  |
|  | 1. Individuals undertook PHIM to achieve optimal health and healthcare outcomes, enable self-advocacy, and manage their health finances. |
|  | 1. Individuals’ situational and psychological attributes converged in unique and dynamic ways to increase the PHIM workload burden and the capabilities required to complete PHIM work. |
| **Tasks** |  |
|  | 1. A three-part classification structure (medical, financial, reference) was used to organize PHIM. |
|  | 1. Five PHIM tasks (i.e., acquire, organize, process, reconcile, and store) reflect the general workflow for an individual PHI document. |
|  | Organizing schemas were personalized t o the individual using five parameters: |
|  | modality preference (person’s preference for digital, paper, or a combination) |
|  | - 1. usage priorities (e.g., person’s immediate health issue, claim dispute, exercise routine, etc.) |
|  | - 1. transportability requirements (e.g., take to a health visit, travel, emergency evacuation, etc. versus paying bills, future tax filing). |
|  | - 1. schema familiarity (e.g., schemas currently used by individuals such as reverse chronological order by provider specialty or name vs. disease or problem, etc.) |
|  | complexity tolerance (e.g., person’s medical situation or level of detail the person wants or can handle) |
|  | 1. The process task primarily entailed extracting, entering and tracking medical and financial PHI on an ongoing basis to support self-care, share PHI with their healthcare providers, and track health finances. |
|  | 1. Reconciling medical billing and insurance payment discrepancies required financial proficiency, while correcting discrepancies in medical PHI remained elusive. |
|  | 1. Five storage dispositions (i.e., active, transportable, archive, discard, backup) were used to file documents based upon the timing, frequency, location and stage of processing. |
| **Tools** |  |
|  | 1. Integration tools were used heavily to: a) provide a comprehensive view of an individual’s medical history and self-care activities, b) match medical expenses with insurance claims and payments, and c) reconcile discrepancies in medical and financial information. |
| **OC^a^** |  |
|  | 1. Attributes of provider and insurer generated PHI increased PHIM workload. Attributes included: a) financial-medical PHI bifurcation, b) multiplicity of players, c) PHI ambiguity and unpredictability, d) rule and regulation bound, and e) reconciliation and dispute burden. |
|  | 1. Multiple, imperfectly-connected, constantly-changing provider and insurer PHI repositories increased the PHIM workload by requiring constant surveillance to download and integrate PHI. |
| **SC^b^** |  |
|  | 1. While providing needed PHIM support, the involvement of personal stakeholders created sensitive interpersonal dynamics and made maintaining information access controls complicated. |
| **PC^c^** |  |
|  | 1. The security of PHI housed in the physical and acquired digital spaces of individuals remained inconclusive even with the use of four PHI protection best practices. |
| ^a^ OC = Organizational Context; ^b^ SC = Social Context; ^c^ PC = Physical Context | |
