# Supplementary material for: A Description of Personal Health Information Management Work With a Spotlight on the Practices of Older Adults: Qualitative e-Delphi Study With Professional Organizers
Source: J Med Internet Res. 2023 Mar 31;25:e42330. doi: 10.2196/42330 (PMC10131782; doi:10.2196/42330)
Supplement: Multimedia Appendix 2 [file jmir_v25i1e42330_app2.docx]

| Multimedia Appendix 2 A description of PHIM activities by task. | |
| --- | --- |
| Task | Description of PHIM activities |
|  |  |
| **Acquire** |  |
|  | Locate, determine how to access, and acquire PHI from patient portals & paper medical record repositories |
|  | Enable multi-person access to acquire, if authorized |
|  | Document PHI sources, individuals authorized to access, portal user IDs and passwords, and medical record requests submitted |
| **Organize** |  |
|  | Adopt a 3-part classification structure to house PHI (i.e., medical, financial and reference) |
|  | Design a personalized filing schema for each PHI classification considering the individual’s: |
|  | - usage priorities (urgency, seriousness) |
|  | - transportability needs |
|  | - preferences for modality (paper, digital or both) |
|  | - placement requirements to insure PHI protection (security and confidentiality) and accessibility (inside and outside of the home) |
|  | - familiarity (preferred schemas, schemas already in use) |
|  | - complexity tolerance (ability to handle) |
|  | Establish document control practices as follows: |
|  | - clearly mark date of service on clinical records |
|  | - number pages to maintain order and ensure completeness |
|  | - label as original or copy to identify duplicates |
|  | Determine privacy & confidentiality preferences for medical and financial PHI |
|  | Design tools^a^ to document, integrate, and track medical and financial PHI considering the preferences, needs and abilities of individual |
| **Process** |  |
|  | Set up PHIM system using classification structure and personalized organizing schemas |
|  | Process and share *medical* PHI using PHI tools^a^ to achieve optimal health care outcomes and support self-advocacy including: |
|  | - compile health histories, medication lists, etc. to share with providers |
|  | - extract, enter and track medical appointments and provider contact information using calendars and contact lists |
|  | - share relevant PHI with providers, friends and family |
|  | Understand and process *financial* PHI to manage health finances including: |
|  | - understand how medical billing and insurance work |
|  | - match services received with bills and track insurance explanation of benefits (EOBs), co-pays, deductibles, etc. |
|  | - pay bills and document payments made and source of payment |
|  | Maintain PHIM system by process PHI on a regular basis using classification structure and personalized organizing schemas |
| **Reconcile** |  |
|  | Document and resolve discrepancies in medical and financial PHI |
|  | Understand inconsistencies and errors in *medical* PHI and work to correct including: |
|  | - identify misdiagnoses or misinterpretations (e.g., in forwarded medical records) - report errors or make corrections in patient portals and track to confirm corrections are made - request updates electronically (e.g., via email or phone messages) and track to confirm corrections |
|  | Understand and follow claim dispute and resolution process for *financial* PHI such as: |
|  | - compare benefit coverage with services received to determine whether a claim needs to be disputed |
|  | - understand bill and claim dispute and filing processes of providers and insurers |
|  | - file claim disputes and track to resolution |
| **Store** |  |
|  | File PHI during and after completion of the process and reconcile tasks using personalized organizing schemas and the following dispositions: |
|  | - active – use for daily care, health finance tracking and PHIM system maintenance |
|  | - transport – carry for medical appointments, emergency care, disaster evacuation, travel |
|  | - archive – store for future use such as taxes, end-of-life, legacy transfer |
|  | - discard – shred paper or delete digital files when no longer needed |
|  | - backup – make a copy of PHI that needs to be preserved and store it in a separate location from the original |
|  | Secure PHI and abide by privacy & confidentiality preferences regardless of disposition or storage location |
| ^a^ Tools are discussed in more detail in the Tools section. | |
