# Supplementary material for: A Description of Personal Health Information Management Work With a Spotlight on the Practices of Older Adults: Qualitative e-Delphi Study With Professional Organizers
Source: J Med Internet Res. 2023 Mar 31;25:e42330. doi: 10.2196/42330 (PMC10131782; doi:10.2196/42330)
Supplement: Multimedia Appendix 3 [file jmir_v25i1e42330_app3.docx]

| Multimedia Appendix 3 A description of tools/technologies used in PHIM work across and within tasks. | |
| --- | --- |
| Task | Tool /technology description |
|  |  |
| **Crosscutting** |  |
|  | **To acquire, process, reconcile and store PHI** |
|  | Computer devices^a^ (desktop, laptop, tablet, phone including type (i.e., Mac or PC), HIPAA compliant software and secure Internet connectivity |
| **Acquire** |  |
|  | Patient portals created by insurers and providers |
|  | Portal access documentation^a^ (user IDs, passwords, instructions, and list of authorized individuals including any limitations) to enable patient and authorized stakeholder access |
|  | Forms authorizing stakeholders to access PHI, description of PHI each individual is authorized to access and who made the request (i.e., patient, doctor, family member) |
|  | Requisitions and release forms for medical record requests |
|  | Digital devices^a^ (cloud, thumb & hard drives, CDs, DVDs) or software^a^ (Box, Evernote, Dropbox, Google, etc.) |
| **Organize** |  |
|  | Personal health information (PHI) acquired from multiple sources |
|  | PHI 3-part classification structure (medical-financial-reference)^a^ |
|  | Personalized filing schema for each classification of PHI that considers 6 parameters (ie, usage priorities, transportability needs, modality preferences, placement requirements, schema familiarity, and complexity tolerance)^a^ |
|  | List of standard document control practices including marking with date of service, numbering pages, marking as original or copy |
| **Process** |  |
|  | **Medical health summary forms** |
|  | Active medical issue/problem form^a^ including treating clinician, related medications, insurance provider, etc. for each medical issue |
|  | Medical history form^a^ including vitals, allergies, immunizations, screenings, lab tests, x-rays, scans, procedures, surgeries, hospitalizations, etc. with dates and clinician contact information |
|  | Family medical history form^a^ with diagnoses, causes of death, age at death |
|  | Medication & supplement form^a^ including dosage, start date, end date/present, renewal info (ordering clinician & pharmacy, refill cycle), when taken, why, and any interactions |
|  | Encounter/visit summary forms^a^ (date, reason for visit, suggested treatment, referrals, next appointment) |
|  | **Medical self-care tools** |
|  | Logs & journals used to track activities, health changes & compliance, including during hospitalization |
|  | Treatment information guidelines or reference documents (e.g., medication side effects/interactions, physical therapy instructions) |
|  | Medication & supplement management materials^a^ (e.g. administration & refill schedule, compliance log, pillboxes) |
|  | **Logistical tools** |
|  | Provider and insurer business cards |
|  | Schedulers and calendars^a^ including alerts and reminders for medical appointments, medication refills, claim dispute deadlines, PHIM tasks, etc. |
|  | Spreadsheets, contact software and address books^a^ to record and track provider and insurer contact information |
|  | Record discipline/specialty, dates seen, tests conducted and problems treated for providers (including pharmacy and ancillary) |
|  | **Health finance tools** |
|  | Spreadsheets^a^ to extract, match, enter and track medical services and charges with insurance coverage, bills, EOBs^b^ & payments |
|  | Resource documents including:   - provider bills, insurance explanation of benefit statements (EOBs), co-pay & co-insurance notices, etc. - payment records (receipts, checks, auto pay orders, etc.) |
|  | Billing flowcharts^a^ |
|  | **PHIM work tools** |
|  | PHIM task list and instructions with alerts and reminders for maintaining PHIM system (i.e., extracting and entering, scanning and filing, referencing, replacing, comparing, etc.) |
|  | Voice recorder to record PHIM instructions for replay to help with memory or visual issues |
| **Reconcile** |  |
|  | Spreadsheets^a^ to document and track to resolution claim disputes and requested medical record changes |
|  | Insurer resource documents such as summaries of changes in benefit coverage, provider network directories, claim and appeal instructions and forms |
| **Store** |  |
|  | Digital devices^a^ (cloud, thumb & hard drives, CDs, DVDs) or software^a^ (Box, Evernote, Dropbox, Google, etc.) |
|  | Binders, folders, dividers/subfolders^a^ |
|  | Business card organizer sleeves^a^, sheet protectors |
|  | File containers^a^ (e.g., boxes, cabinets, drawers) |
|  | Scanner to digitize PHI |
|  | Shredder to destroy PHI |
| ^a^  Tools used to integrate PHI from multiple sources  ^b^ EOBs = Explanation of Benefit statements | |
