# Supplementary material for: A Description of Personal Health Information Management Work With a Spotlight on the Practices of Older Adults: Qualitative e-Delphi Study With Professional Organizers
Source: J Med Internet Res. 2023 Mar 31;25:e42330. doi: 10.2196/42330 (PMC10131782; doi:10.2196/42330)
Supplement: Multimedia Appendix 4 [file jmir_v25i1e42330_app4.docx]

| Multimedia Appendix 4 Representative quotations for lists of integration tools used in the process and reconcile task. | |
| --- | --- |
| Representative quotations (R#Q#^a^) | Tool type |
|  |  |
| ***I encourage* [individuals] *to build their own …*** |  |
| *… personal history of preventive actions (immunizations, screening test dates and results), …* | Medical (history) |
| *… allergies (medical and other), …* | Medical (current) |
| *… diagnoses (acute and chronic), …* | Medical (current & history) |
| *… surgeries (dates), …* | Medical (history) |
| *… prescriptions, …* | Medical (current) |
| *… as well as a family history of diagnoses, causes of death and age at death.* (R1Q4) | Medical (family history) |
| ***Keep* [the following]:** |  |
| *1) a dedicated master health calendar; note doctor's name, purpose of visit, tests performed; this provides a memory cue when a bill is received for something not recognized by the patient …* | Medical-Finance (calendar) |
| *… 2) a master doctor list: by specialty; name, address, phone, fax, receptionist name, PA's name, nurse's name; …* | Logistical (contact list) |
| *… 3) a master prescription list [with] name, dosage, start date, end date, renewal info; …* | Medical (current) |
| *… 4) file for each routine test (blood, MRIs, etc); …* | Medical (history) |
| *… 5) medical event history for surgeries, hospitalizations, procedures.* … | Medical (history) |
| *… In the hospital, (if able) have a notebook and each day record every doctor that 'stops in', medications, tests etc.* (R1Q4) | Medical (current & self-care) |
| ***Keep an:*** |  |
| *Excel chart for health history with labs, body measurements such as weight, BP, X-rays and scans.* (R1Q4) | Medical (history) |
| ***I have often found it useful to…*** |  |
| *… develop a flow chart of how billing occurs. A* [person] *may have some providers who directly bill insurance, others who require the patient to pay and then file. A Health Savings Account may be involved. For* [the person I’m assisting] *(and for myself to understand their situation), I often need to map out the possible paths to bill resolution and what intermediate action files will be needed.* (R2Q4) | Finances (flowchart) |
| ***Having …*** |  |
| *… a journal / calendar for only medical appointments for easy tracking.* (R1Q4) | Logistics (calendar) |
| ***Organizing their prescriptions and supplements*** |  |
| *Many* [individuals] *also need help in organizing their prescriptions and supplements - in terms of tracking dosages to take/when, setting up a refill schedule, and maintaining an Rx log for relatives/health care home workers and providers as well as just organizing the physical pill bottles.* (R2Q4) | Medical (self-care) |
| ***One 'History' binder with clearly labeled sections for:*** |  |
| *… a) Doctors, pharmacy - contact info; …* | Logistical (contact) |
| *… b) Prescriptions - what, when, why, how much, interactions; …* | Medical (current) |
| *… c) Surgeries, illnesses - what, when; …* | Medical (history) |
| *… d) Physical or occupational therapy - current & prior.* (R1Q4) | Medical (current & history) |
| ***One 'Billing' binder for billing details & summary with sections for:*** |  |
| *… a) Medicare - as Medicare stmts arrive, match up details to summary; …* | Finance (match) |
| *… b) Supplemental Insurance - Medigap; …* |  |
| *… c) Prescriptions – receipts; …* |  |
| *… d) Paid bills - x-ref to form of pymt.* (R1Q4) |  |
| ***I also recommend …*** |  |
| *… to capture billing data by category (doc vs prescrip vs hospital) for tax purposes.* (R1Q4) | Finance (track) |
| ***Keep separate folders for …*** |  |
| *… different insurance companies (i.e.- Medicare, secondary insurance, prescription drug info). Organize chronologically and when new info comes in cross check to be sure all available payment options have been used. Info then can be shredded or scanned or filed as needed. …* | Finance (track) |
| *… Excel chart for above allows for additional notations on actions taken, calls made, etc.* (R1Q4) | Finance (reconcile) |
| ^a^ R#Q# = Specifies the Delphi Round number and Question number for quotation. | |
