# Supplementary material for: A Description of Personal Health Information Management Work With a Spotlight on the Practices of Older Adults: Qualitative e-Delphi Study With Professional Organizers
Source: J Med Internet Res. 2023 Mar 31;25:e42330. doi: 10.2196/42330 (PMC10131782; doi:10.2196/42330)
Supplement: Multimedia Appendix 5 [file jmir_v25i1e42330_app5.docx]

| Multimedia Appendix 5 Representative quotations for distinctive attributes of PHI generated by insurers and providers. | |
| --- | --- |
| Attributes | Representative Quotations (R#Q#^a^) |
|  |  |
| **PHI bifurcation** |  |
|  | *The financial/medical issue is unique to* [PHI] *…* (R2Q6) |
| **Multiplicity of players** |  |
|  | [continued from quotation above] *… as is the number of players (doctor, hospital, labs, therapist, insurance company (1 or more))* [unique to PHI]. (R2Q6) |
| **PHI ambiguity and unpredictability** |  |
|  | *Often* [PHI] *is difficult to give a clear "label." An electric bill is pretty straight forward-pay it and let it go. A statement from Medicare may not be so easily handled. Is it* [for your] *information* [only]*? a bill? a notice that the secondary insurer has also been billed?* (R2Q5) [Lack of clarity] |
|  | *Better transparency of communication between medical providers, medical billing, and insurance companies so that* [individuals] *can view the path of their financial information, billing status, coverage status, etc.* [is needed]*.* (R3Q1) [Lack of transparency] |
|  | *Many* [individuals] *are assuming that their providers are in communication with each other. But in many cases, they are not.* (R3Q4) [Lack of coordination] |
|  | *Education in terminology related to the field … Medical Terminology – basic terms* [and] *Insurance & Social Security 101 – basic terms, practices* [is needed to assist with PHIM]*.* (R2Q8) [Dual terminologies] |
|  | [Financial] *information is coming from different sources and at different times. So it’s not like organizing financial paperwork where you expect a bill every month.* (R2Q6) [Unpredictability] |
| **Rule and regulation bound** |  |
|  | *There are rules regarding what is helpful and/or necessary with regard to medical and financial information. … Most non-medical/financial paperwork organizing is guided less by rules and more by functionality and by the personal/customized needs of the* [person] *and other family stakeholders … [Non-medical/financial information may be organized] even in counter-intuitive ways, to make the* [person] *happy, because efficiency may be at odds with a concern for aesthetics.* (R2Q6) |
|  | *With financial and medical information organizing, it's important to clarify for* [individuals] *the financial and accessibility issues (deadlines, fees, credit consequences, lost time, lack of access for others, etc.) that occur when financial/health information is not organized properly.* (R2Q6) |
| **Reconciliation and dispute burden** |  |
|  | *A knowledge of how medical billing works - how to read an EOB, a provider bill, a Medicare statement. Know what a primary and secondary insurer is. Understand how Medicare works.* (R3Q8) |
|  | [Understand] *diagnosis codes …* [and] *understanding the process of how insurance is filed* [is a challenge for individuals]*.* (R1Q2) |
| ^a^ R#Q# = Specifies the Round number and Question number for quotation | |
