# Supplementary material for: A Description of Personal Health Information Management Work With a Spotlight on the Practices of Older Adults: Qualitative e-Delphi Study With Professional Organizers
Source: J Med Internet Res. 2023 Mar 31;25:e42330. doi: 10.2196/42330 (PMC10131782; doi:10.2196/42330)
Supplement: Multimedia Appendix 6 [file jmir_v25i1e42330_app6.docx]

| Multimedia Appendix 6 Representative quotations for attributes of provider and insurer PHI repositories. | |
| --- | --- |
| Attributes | Representative quotations (R#Q#^a^) |
|  |  |
| **Multiple, Imperfectly-Connected** |  |
|  | *For instance, a* [person] *may have an immediate health issue and need to organize test and lab information for the next consultant, especially if they are seeking a 2nd opinion in some other (imperfectly-connected) care system. But the* [person] *will also need to keep track of tests and labs to confirm/contest billing later.* (R2Q1) |
|  | *Electronic would be best. Being able to move information securely from one physician to another. Currently you can't download and then upload to another clinic if it's out of network* [which is a barrier to assisting with PHIM]*.* (R1Q3) |
| **Constantly-Changing** |  |
|  | *Changing health care systems* [are a challenge]*. What you know about the relationship of different providers is constantly changing along with their* [Health Information Management Systems]*.* (R2Q2-3) |
| ^a^ R#Q# = Specifies the Delphi Round number and Question number for quotation. | |
