# Supplementary material for: A Description of Personal Health Information Management Work With a Spotlight on the Practices of Older Adults: Qualitative e-Delphi Study With Professional Organizers
Source: J Med Internet Res. 2023 Mar 31;25:e42330. doi: 10.2196/42330 (PMC10131782; doi:10.2196/42330)
Supplement: Multimedia Appendix 7 [file jmir_v25i1e42330_app7.docx]

| Multimedia Appendix 7 Representative quotations that demonstrate how personal stakeholder involvement interacts with other Patient Work System components to create sensitive interpersonal dynamics. | |
| --- | --- |
| Primary SC  Interaction^a^ | Representative quotations (R#Q#^b^) |
|  |  |
| **Person** |  |
|  | **PHIM assistance** |
|  | [Are] *family members able to assist* [with PHIM]*?* (R1Q1) |
|  | [Is PHIM] *assistance from a support group … friend or caregiver possible?* (R2Q1) |
|  | **PHIM privacy** |
|  | *Many* [individuals] *feel shame about their medical issues and they feel embarrassed to let someone see their private medical papers.* (R1Q3) |
|  | *[A person] not willing to discuss* [PHIM] *although his/her family members know it needs to be done* [is a PHIM challenge]. (R1Q2) |
|  | *Health information is more personal and private and clients may wish to keep certain medical conditions hidden.* (R3Q5) |
|  | **Roles and goals (conflicting)** |
|  | *Who is initiating …* [the request for PHIM assistance]*? A “helpful” parent, spouse or other force?* [A person may be] *dependent on others to maintain or assist in keeping information without taking responsibility to maintain their own information.* (R2Q2-3) |
|  | *Seniors may be facing … the emotional and physical demands of being either caretaker or care receiver as well as potential downsizing (either self-determined or not) and inter-generational struggles around who's "in charge."* (R2Q5) |
|  | *Multiple family members with conflicting goals and levels of involvement* [are barriers for helping with PHIM]. (R1Q3) |
| **Tasks** |  |
|  | **Access (earning trust)** |
|  | *Earning the* [person’s] *trust to be comfortable discussing personal information and ensuring its confidentiality* [is a barrier for helping with PHIM]. [Some people] *wish to keep medical conditions/ medications private.* (R1Q3) |
|  | **Process (medications)** |
|  | *Many* [individuals] *also need help in organizing their prescriptions and supplements - in terms of tracking dosages to take/when, setting up a refill schedule, and maintaining an Rx log for relatives/health care home workers and providers as well as just organizing the physical pill bottles.* (R2Q4) |
|  | *Once you've “organized” meds & supplements,* [the person] *may want you to fill daily pill doses for example. HUGE LIABILITY issue.* (R3Q4) |
| ^a^ Primary interactions between Social Context (SC) and other Patient Work System component (i.e., Person, Tasks, Tools, Organizational Context (OC)).  ^b^ R#Q# = Specified the Delphi Round number and Question number for quotation. | |
