# Supplementary material for: A Description of Personal Health Information Management Work With a Spotlight on the Practices of Older Adults: Qualitative e-Delphi Study With Professional Organizers
Source: J Med Internet Res. 2023 Mar 31;25:e42330. doi: 10.2196/42330 (PMC10131782; doi:10.2196/42330)
Supplement: Multimedia Appendix 8 [file jmir_v25i1e42330_app8.docx]

| Multimedia Appendix 8 Representative quotations that demonstrate how personal stakeholder involvement interacts with other Patient Work System components to complicate maintaining information controls. | |
| --- | --- |
| Primary SC interaction^a^ | Representative quotations (R#Q#^b^) |
|  |  |
| **Person** |  |
|  | **Multiple roles and people** |
|  | *A* [person] *may also be responsible for health information for other family members, multiplying the information control issues.* (R1Q2) |
|  | *How many people in the* [person’s] *household need to maintain information?* (R1Q1) |
|  | *A* [person] *may be a caregiver for others, but also a patient themselves.* (R2Q2-3) |
|  | **Advocacy role** |
|  | *Understand … who may assist with medical decisions.* (R1Q3) |
| **Tasks** |  |
|  | **Process (maintain, share) and Store (active, transport)** |
|  | *--Who is responsible for recording/maintaining the information?*  *--Who is responsible for accessing the information? Under what circumstances (at home, at a care facility, at medical appointments, etc.) must the information be accessed?* (R1Q1) |
|  | **Process (access, share)** |
|  | *Who is data being maintained BY and SHARED WITH (accessibility).* (R2Q1) |
|  | **Process (access, maintain)** |
|  | *Who will be maintaining the health information organization system once implemented? If it is various individuals - the* [person]*, spouse, adult children, caretakers, etc. - there needs to be a buy-in or at least an education to all on how to use the system.* (R2Q1) |
|  | **Reconcile (access, advocate)** |
|  | *Who has authority to be an advocate if required … such as insurance payments and contacting insurance companies and doctors.* (R1Q3) |
|  | **Store (access, advocate)** |
|  | *Consider who can ACCESS as well as advocate/authorize and what happens if there's no documented decision regarding which persons (second spouses, step-children, etc.) can have access.* (R2Q2-3) |
| ^a^ Primary interaction between Social Context (SC) and named Patient Work System component (i.e., Person, Task, Tool, Physical Context, Organizational Context).  ^b^ R#Q# = Specified the Delphi Round number and Question number for quotation. | |
