# Supplementary material for: A Description of Personal Health Information Management Work With a Spotlight on the Practices of Older Adults: Qualitative e-Delphi Study With Professional Organizers
Source: J Med Internet Res. 2023 Mar 31;25:e42330. doi: 10.2196/42330 (PMC10131782; doi:10.2196/42330)
Supplement: Multimedia Appendix 9 [file jmir_v25i1e42330_app9.docx]

| Multimedia Appendix 9 Representative quotations that demonstrate how physical context interacts with other Patient Work System components to influence the protection of PHI. | | |
| --- | --- | --- |
| Primary PC  Interactions^a^ | Representative quotations (R#Q#^b^) |  |
|  |  |  |
| **PC** |  |  |
|  | **Secure PHI (physical and digital spaces)** |  |
|  | *Keeping documents secure if taken outside of [a person’s] home and/or digitally kept* [is a PHIM best practice]. (R1Q4) |  |
|  | *Secure data if located outside of* [the person's] *home or on* [the person’s] *computer* [is a PHIM best practice]. (R3Q4) |  |
| **Person** |  |  |
|  | **Expectations (privacy, security & confidentiality)** |  |
|  | *Earning the* [person’s] *trust to be comfortable discussing personal information and ensuring its confidentiality [is a barrier for assisting with PHIM].* [Some individuals] *wish to keep medical conditions/ medications private.* (R1Q3) |  |
|  | *Assuring* [the person] *that all work is strictly confidential and adhering to that* [is a PHIM best practice]. (R1Q4) |  |
|  | *Health information can be very sensitive information, so extra care must be taken to maintain security and confidentiality.* (R2Q5) |  |
|  | *Health information is more personal and private and* [individuals] *may wish to keep certain medical conditions hidden.* (R3Q6) |  |
|  | **Authorized access (whom, how often, where, how, tasks)** |  |
|  | *--Who is responsible for recording/maintaining the information?*  *--Who is responsible for accessing the information? Under what circumstances (at home, at a care facility, at medical appointments, etc.) must the information be accessed?* (R1Q1) |  |
|  | *I kept adding “by whom” to* [the following bullets]: (R2Q1)   - *How often does the health information need to be referenced and by whom?* - *How will the information be accessed and by whom? Digitally or through a paper copy?* - *How will the health information be used and by whom?* |  |
|  | *Consider who can ACCESS as well as advocate/authorize and what happens if there's no documented decision regarding which persons (second spouses, step-children, etc.) can have access.* (R2Q2-3) |  |
| **Person***  **Tools** |  |  |
|  | **Security of digital software, apps (knowledge, trust)** |  |
|  | *How comfortable are the individuals in the* [person’s] *household with technology? If applicable, what technology solutions does the* [person’s] *household already use for maintaining information (Evernote, Dropbox, Google, etc.)?* (R1Q1) |  |
|  | *Some* [individuals] *are unclear about how to scan and keep records securely online or not trusting of the security of doing so.* (R1Q2) |  |
|  | *I … scan all … health records and keep them in Box.com (more secure than Dropbox and HIPAA Compliant)* [as a PHIM best practice]*.* (R1Q4) |  |
|  | *Sometimes, a* [person] *wants to use Evernote; but although it is secure, it does not meet HIPAA-level security, and the* [person] *must a) be made aware of this and b) taught how to use note-level encryption.* (R2Q1) |  |
|  | **Security of cloud storage** |  |
|  | *Scan info into specific sites/apps that keep health records securely in the cloud or on their site* [is a best PHIM practice]. (R1Q4) |  |
|  | *Scanning all documents and storing in separate digital folders on a HIPAA compliant cloud* [is a best practice]*.* [R1Q4] |  |
|  | *I often suggest scanning paperwork and saving to cloud storage, so the information can be accessed remotely, if necessary; however, some* [individuals] *don't trust that it will be held securely.* (R2Q6) |  |
|  | **HIPAA Training** |  |
|  | *Anyone dealing with medical information needs to be HIPAA trained and confidentiality should be addressed in the* [PHIM assistance] *contract.* (R2Q6) |  |
|  | *Know about HIPAA laws, releases,* [and] *secure communications* [when working with PHI]. (R3Q8) |  |
| **Tools** |  |  |
|  | **Environmental hazards (paper)** |  |
|  | *The placement of the storage system and environmental concerns should be considered. Systems where animals or small children can access/destroy documents, paper files kept near walls with plumbing that could burst or near windows or fans that could blow unfiled documents around are all concerns.* (R2Q2-3) |  |
|  | **Backup (paper and digital)** |  |
|  | *Organizing health information has the added concern of privacy and security of information, so that needs to be addressed in the access, storage and data backup considerations.* (R1Q1) |  |
|  | *Documentation (paper &/or electronic) of healthcare info in one secure, repository -- with secure backup stored elsewhere* [is a PHIM best practice]. (R1Q4) |  |
|  | *Ensuring secure digital back up of health information* [is a PHIM best practice]. (R1Q4) |  |
|  | **Confidential (shredding)** |  |
|  | [To] *shred papers with confidential personal info* [is a PHIM best practice]. (R1Q4) |  |
|  | **Privacy, trust (visual cues)** |  |
|  | *Privacy and trust are so important in medical record organizing more so than organizing objects. For the visual* [individuals]*, is it easy to organize objects so they can see them, find them, and use them. In medical information organizing, it is harder to design the system visually while keeping it protected and private so they can find it and use it.* (R2Q6) |  |
| **OC*Tasks** |  |  |
|  | **No provider to provider digital sharing** |  |
|  | *Electronic would be best. Being able to move information securely from one physician to another. Currently you can't download and then upload to another clinic if it's out of network* [which is a barrier to assisting with PHIM]*.* (R1Q3) |  |
| ^a^ Primary interactions with Physical Context (PC) and other Patient Work System components (i.e., Person, Tasks, Tools, Social Context (SC), Organizational Context (OC).  ^b^ R#Q# = Specified the Delphi Round number and Question number for quotation. | |  |
